# Multilevel thresholding satellite image segmentation using chaotic coronavirus optimization algorithm with hybrid fitness function

**DOI:** 10.1007/s00521-022-07718-z

**Published:** 2022-09-23

**Authors:** Khalid M. Hosny, Asmaa M. Khalid, Hanaa M. Hamza, Seyedali Mirjalili

**Affiliations:** 1 Department of Information Technology, Faculty of Computers and Informatics, Zagazig University, Zagazig, 44519 Egypt; 2 Centre for Artificial Intelligence Research and Optimisation, Torrens University Australia, Fortitude Valley, Brisbane, QLD 4006 Australia

**Keywords:** Image segmentation, Optimization, Thresholding, Metaheuristic, Satellite

## Abstract

Image segmentation is a critical step in digital image processing applications. One of the most preferred methods for image segmentation is multilevel thresholding, in which a set of threshold values is determined to divide an image into different classes. However, the computational complexity increases when the required thresholds are high. Therefore, this paper introduces a modified Coronavirus Optimization algorithm for image segmentation. In the proposed algorithm, the chaotic map concept is added to the initialization step of the naive algorithm to increase the diversity of solutions. A hybrid of the two commonly used methods, Otsu’s and Kapur’s entropy, is applied to form a new fitness function to determine the optimum threshold values. The proposed algorithm is evaluated using two different datasets, including six benchmarks and six satellite images. Various evaluation metrics are used to measure the quality of the segmented images using the proposed algorithm, such as mean square error, peak signal-to-noise ratio, Structural Similarity Index, Feature Similarity Index, and Normalized Correlation Coefficient. Additionally, the best fitness values are calculated to demonstrate the proposed method's ability to find the optimum solution. The obtained results are compared to eleven powerful and recent metaheuristics and prove the superiority of the proposed algorithm in the image segmentation problem.

## Introduction

Digital image processing is manipulating digital images through algorithms using digital computers for many purposes, such as image enhancement, image compression, and extracting useful information [1]. Image segmentation is a crucial process in most digital image processing tasks. It isolates the region of interest from the scene [2]. Image segmentation has been successfully applied to several fields, such as image denoising [3], medical image diagnosis [4], and satellite image segmentation [5]. In the literature, several techniques have been proposed for image segmentation. These techniques can be categorized as edge detection-based segmentation [6], clustering-based segmentation [7], and thresholding-based segmentation [8]. Thresholding-based segmentation is considered the most popular technique because of its simplicity and efficiency. In thresholding-based segmentation, the histogram information is extracted from the grayscale image and is used to determine threshold values to separate image pixels into different classes [9]. When one threshold value is needed, it is referred to as bi-level thresholding, in which the image is segmented into only two regions.

Multilevel thresholding is more appropriate in images containing many objects with fine details and complex backgrounds because bi-level thresholding fails to distinguish these objects correctly. After all, it divides the image into only two regions [10]. On the other hand, multilevel thresholding involves using more than one threshold to segment the image into several regions [11]. The thresholding process aims to find the best threshold values that precisely determine the image segments. Otsu [12] and Kapur [13] methods are considered the most popular strategies for determining the optimal thresholds. Otsu's method maximizes the variance between classes, while Kapur's method maximizes the histogram entropy to measure homogeneity between segmented regions.

Over the last few years, Swarm intelligence has been extensively applied to solve multilevel thresholding image segmentation problems [14]. Many algorithms have been proposed for satellite image segmentation, such as a modified version of an artificial bee colony (MABC) proposed by Bhandari et al. [15]. The results reveal that MABC has more computational efficiency and accuracy than the standard ABC. For RGB histogram-based color satellite image segmentation, a multi-strategy Emperor Penguin Optimizer (MSEPO) is proposed by Heming et al. [16]. The results showed that the MSEPO algorithm had superior performance, especially for the high dimensional segmentation of complex satellite images. The proposed hybrid Grasshopper Optimization Algorithm and Differential Evolution (GOA-jDE) has been proposed by Heming et al. [17]. The superiority of the proposed algorithm is illustrated in terms of different metrics such as peak signal-to-noise ratio (PSNR), structural similarity index (SSIM), feature similarity index (FSIM), and standard deviation (STD), convergence performance, and computation time. Many other algorithms for satellite image segmentation have been proposed in [18–21].

Several algorithms have been proposed in medical images, such as ant colony optimization with Cauchy and greedy levy mutations for COVID X-ray images segmentation [22]. Bandyopadhyay et al. [4] proposed an altruistic Harris Hawks’ optimization algorithm to segment brain MRI images. This algorithm combines the chaotic initialization, the concept of altruism, and a hybrid objective function, where the results show superior searchability and convergence speed performance. Also, Abualigah et al. [23] proposed an evolutionary arithmetic optimization algorithm for COVID-19 CT image segmentation. According to the experimental results, the proposed algorithm produces higher-quality solutions than other comparisons. Other techniques for medical image segmentation are proposed in [24–27].

In recent years, chaotic maps were incorporated into the swarm intelligence algorithms to increase the diversity of solutions and avoid falling into local optimum [28]. Hongwei et al. [29] proposed a Chaos-enhanced moth-flame optimization (MFO) algorithm for global optimization. The statistical results demonstrate that the appropriate chaotic map (singer map) embedded in the appropriate component of MFO can significantly improve the performance of MFO. [30], two different chaotic maps were incorporated into the original elephant herding optimization algorithm. Test results proved that the proposed chaotic elephant herding optimization algorithm performs better and obtains better results. Aggarwal et al. [31] used the chaotic sequence to initialize the social spider optimization algorithm, enhancing its performance. Many other researchers have embedded the chaotic concept into their native algorithms to enhance their search ability [32–36].

Coronavirus Optimization Algorithm (COVIDOA) is a recent metaheuristic inspired by the replication lifecycle of Coronavirus [37]. COVIDOA has three main phases: Virus Entry, Virus Replication, and Virus mutation. Coronavirus uses frameshifting [38–40] to make new virus copies in the Replication phase. Frameshifting produces many viral proteins combined to form new virus particles as many new particles are created, and many human cells are damaged. In addition, the virus uses mutation techniques to escape from the human immunity system. COVIDOA has been applied to many benchmark test functions and real-world problems and showed superior performance. Its advantages include a good balance between exploration and exploitation and high convergence speed.

This paper introduces the chaotic map concept into the novel Coronavirus Disease Optimization Algorithm (COVIDOA) to increase the diversity of solutions. The proposed algorithm is applied to solve the multilevel thresholding image segmentation problem of satellite images and a set of benchmark images. The proposed algorithm used a hybrid fitness function to find the optimum threshold values by adding weights to the Otsu and Kapur methods. The results showed that using the hybrid fitness function and adding the chaotic maps yields significantly better results than the other proposed algorithms. The motivation for using modified COVIDOA for satellite image segmentation is as follows: The No Free Lunch (NFL) [41] theorem demonstrates that no single algorithm performs best for all optimization problems; this encouraged us to use a modified version of the recent COVIDOA to solve image segmentation problem.

Additionally, the basic and the binary versions of COVIDOA have performed much better in solving many benchmark and real-world problems [37, 42]; real world it can be assumed that, if the basic version is improved, it can also perform well in solving complex optimization problems such as multilevel thresholding problem. It is observed from the literature work that most of the authors used either the Otsu method or Kapur’s entropy as a fitness function for solving multilevel thresholding problems, which encouraged the authors to use a new hybrid fitness function with a modified COVIDOA to achieve better results in solving the multilevel thresholding image segmentation problem.

The main contributions of this paper can be summarized as follows:

1. The chaotic logistic map is used to initialize COVIDOA to increase the diversity of solutions.
2. A new hybrid fitness function is used for finding the optimum thresholds by assigning weights to the Otsu and Kapur methods.
3. The superiority of the proposed algorithm is validated by applying it to six satellite and six benchmark images.
4. The proposed method for image segmentation results is compared with many state-of-the-art algorithms focusing on the recently proposed metaheuristics.
5. Several measures are used to evaluate the performance of the proposed algorithm in solving multilevel thresholding problems, such as best fitness value, MSE, PSNR, SSIM, FSIM, and NCC, and conducting the Wilcoxon rank-sum test to prove the efficiency of the proposed algorithm.

This paper is organized as follows: Sect. 2 provides a brief overview of multilevel thresholding techniques such as Otsu’s method, Kapur’s entropy, and the hybrid of the two objective functions. The proposed Coronavirus disease optimization with chaotic map initialization for multilevel thresholding is discussed in Sect. 3. The datasets, parameter setting, performance metrics, and experimental results are discussed in Sect. 4. Finally, conclusions and future work are given in Sect. 5.

## Multilevel thresholding

Image thresholding is a simple and effective method for splitting the image into regions to make the image easier to analyze. Setting the threshold value *t* is based on the pixel intensity of the image, where pixels whose intensity values below *t* are assigned to region 1, and the other pixels are assigned to region 2 [43]. If only one threshold value is needed, this is known as bi-level thresholding, where the image is divided into two regions.

1 $$ \begin{aligned} & {\text{pixel}}_{i,j} \in R_{1} \quad {\text{if}}\quad 0 \le {\text{pixel}}_{i,j} < t, \\ & {\text{pixel}}_{i,j} \in R_{2} \quad {\text{if}}\quad t \le {\text{pixel}}_{i,j} < L - 1, \\ \end{aligned} $$

where ${\text{pixel}}_{i,j}$ refers to the gray level at the (*i*, *j*)th pixel, *t* is the value of the threshold, $R_{1}$ and $R_{2}$ refer to region 1 and region 2, respectively, and $L$ refers to maximum intensity level.

On the other hand, multilevel thresholding partitions the image into several distinct regions using more than one threshold value as follows:

2 $$ \begin{aligned} & {\text{pixel}}_{i,j} \in R_{1} \quad {\text{if}}\quad 0 \le {\text{pixel}}_{i,j} < t_{1} , \\ & {\text{pixel}}_{i,j} \in R_{2} \quad {\text{if}}\quad t_{1} \le {\text{pixel}}_{i,j} < t_{2} , \\ & {\text{pixel}}_{i,j} \in R_{j} \quad {\text{if}}\quad t_{j} \le {\text{pixel}}_{i,j} < t_{j + 1} , \\ & {\text{pixel}}_{i,j} \in R_{k} \quad {\text{if}}\quad t_{k} \le {\text{pixel}}_{i,j} < L - 1, \\ \end{aligned} $$

where $\left\{ {t_{1} ,t_{2} , \ldots , t_{k} } \right\}$ represents a vector of different threshold values.

**Fig. 1:**
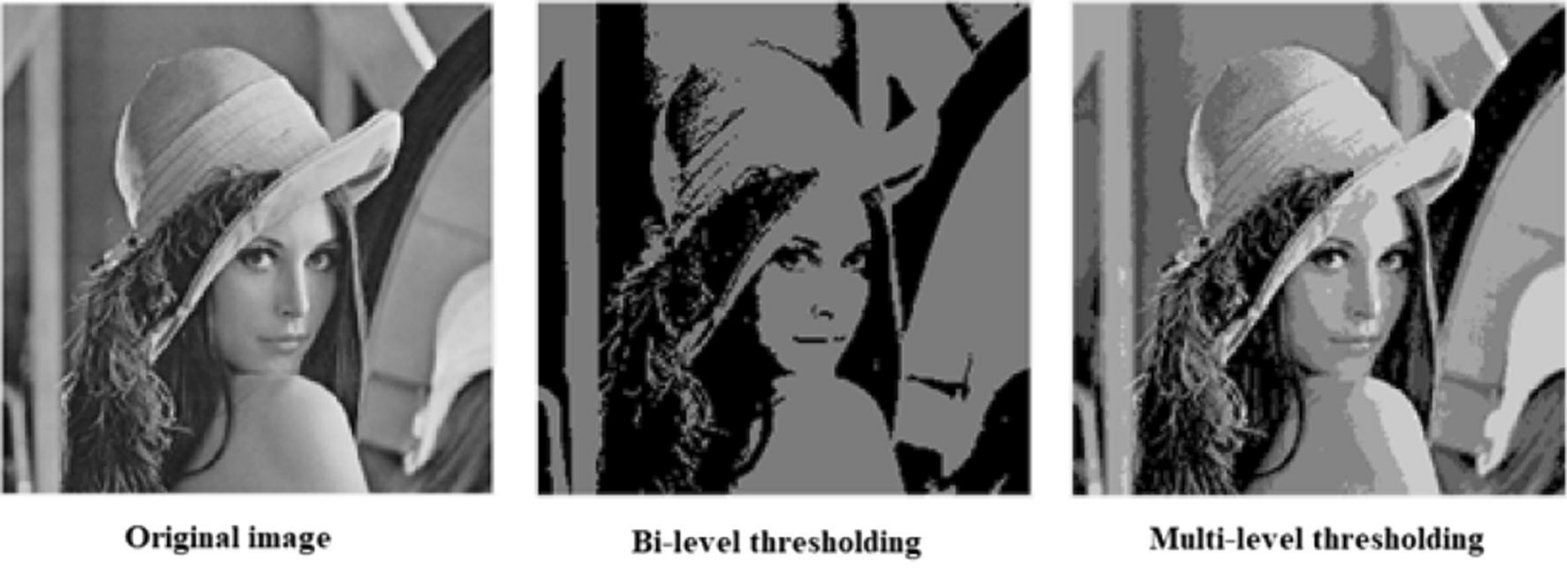
Bi-level and multilevel thresholding

The result of applying bi-level versus multilevel thresholding on the Lena image is shown in Fig. 1.

The optimal threshold values can be obtained by maximizing a fitness function. Otsu’s method and Kapur’s entropy are two popular techniques used in thresholding. Each technique proposes a different fitness function that must be maximized to obtain the optimal threshold values. The two techniques are briefly described in the following subsections.

### Otsu’s method

Otsu is a thresholding method that selects the optimal threshold by maximizing the variance value between different classes [12]. Assume that we have *L* intensity levels in a grayscale image, where *L* = 256 and a vector *V* of *k* − 1 thresholds are used to segment the image into *K* regions as in Eq. (2), where *V* = [th_1_, th_2_, …, th_*k* − 1_]. Then the best threshold is obtained by maximizing the Otsu’s fitness function as follows:

3 $$ F_{{{\text{ostu}}}} (V) = \max \left( {\sigma_{b}^{2} (V)} \right) $$

where $\sigma_{b}^{2}$ represents the between-class variance which can be expressed as follows:

4 $$ \sigma_{b}^{2} = \mathop \sum \limits_{k = 0}^{K} \omega_{k} \cdot \left( {\mu_{k} - \mu_{T} } \right)^{2} $$

where $\omega_{k}$ is the cumulative probability for region *R*_*k*_, $\mu_{k}$ is the average intensity in region *R*_*k*_ and $\mu_{T}$ is the average intensity for the whole image as follows:

5 $$ \omega_{k} = \mathop \sum \limits_{{i \in R_{k} }} P_{i} ,\quad \mu_{k} = \mathop \sum \limits_{{i \in R_{k} }} \frac{{i \cdot P_{i} }}{{\omega_{k} }},\quad \mu_{k} = \mathop \sum \limits_{i = 0}^{L - 1} i \cdot P_{i} $$

where $$ P_{i}$$ is the probability of gray level *i,* which can be represented as follows:

6 $$ P_{i} = \frac{{f_{i} }}{{\mathop \sum \nolimits_{i = 0}^{L - 1} f_{i} }} $$

where *f*_*i*_ is the frequency of gray level *i*.

### Kapur’s entropy method

Image entropy represents the compactness and separateness between image classes [13]. The Kapur method is another widely used thresholding method that aims to find the optimal threshold value by maximizing the Kapur’s entropy as follows:

7 $$ {\text{th}}^{*} = \max (F_{{{\text{kapur}}}} ({\text{th}})) $$

where

$$ \begin{aligned} & F_{{{\text{kapur}}}} ({\text{th}}) = A_{0} + A_{1} , \\ & A_{0} = - \mathop \sum \limits_{i = 0}^{{{\text{th}} - 1}} \frac{{P_{i} }}{{\omega_{0} }}\ln \frac{{P_{i} }}{{\omega_{0} }}, \\ & A_{1} = - \mathop \sum \limits_{{i = {\text{th}}}}^{L - 1} \frac{{P_{i} }}{{\omega_{1} }}\ln \frac{{P_{i} }}{{\omega_{1} }}, \\ & \omega_{0} = \mathop \sum \limits_{i = 0}^{{{\text{th}} - 1}} P_{i} ,\quad \omega_{1} = \mathop \sum \limits_{{i = {\text{th}}}}^{L - 1} P_{i} , \\ \end{aligned} $$

where ${P}_{i}$ is described in Eq. (6).

For multilevel thresholding, Kapur’s method can be defined as follows:

8 $$ \begin{aligned} & F_{{{\text{kapur}}}} \left( V \right) = A_{0} + A_{1} + \cdots + A_{k - 1} \\ & A_{0} = - \mathop \sum \limits_{i = 0}^{{{\text{th}}_{1} - 1}} \frac{{P_{i} }}{{\omega_{0} }}\ln \frac{{P_{i} }}{{\omega_{0} }},\quad \omega_{0} = \mathop \sum \limits_{i = 0}^{{{\text{th}}_{1} - 1}} P_{i} \\ & A_{1} = - \mathop \sum \limits_{{i = {\text{th}}_{1} }}^{{{\text{th}}_{2} - 1}} \frac{{P_{i} }}{{\omega_{1} }}\ln \frac{{P_{i} }}{{\omega_{1} }},\quad \omega_{1} = \mathop \sum \limits_{{i = {\text{th}}_{1} }}^{{{\text{th}}_{2} - 1}} P_{i} \\ & A_{2} = - \mathop \sum \limits_{{i = {\text{th}}_{2} }}^{{{\text{th}}_{3} - 1}} \frac{{P_{i} }}{{\omega_{2} }}\ln \frac{{P_{i} }}{{\omega_{2} }},\quad \omega_{2} = \mathop \sum \limits_{{i = {\text{th}}_{2} }}^{{{\text{th}}_{3} - 1}} P_{i} \\ & A_{k - 1} = - \mathop \sum \limits_{{i = {\text{th}}_{k - 1} }}^{L - 1} \frac{{P_{i} }}{{\omega_{k - 1} }}\ln \frac{{P_{i} }}{{\omega_{k - 1} }},\quad \omega_{2} = \mathop \sum \limits_{{i = {\text{th}}_{k - 1} }}^{L - 1} P_{i} \\ \end{aligned} $$

The vector *V* refers to thresholds to be determined.

### Hybrid fitness function

A hybrid fitness function calculates COVID solutions' fitness in image segmentation problems. This hybrid function is formulated by assigning weights to Otsu and Kapur functions in Eq. 9.

9 $$ F_{{{\text{hybrid}}}} = aF_{{{\text{Otsu}}}} + bF_{{{\text{Kapur}}}} $$

where *a* and *b* $\in$ [0, 1] are weights associated with the two fitness functions and *a* + *b* = 1. The proposed hybrid fitness function optimizes Otsu and Kapur methods simultaneously and performs more efficiently.

## Coronavirus disease optimization algorithm

**Fig. 2:**
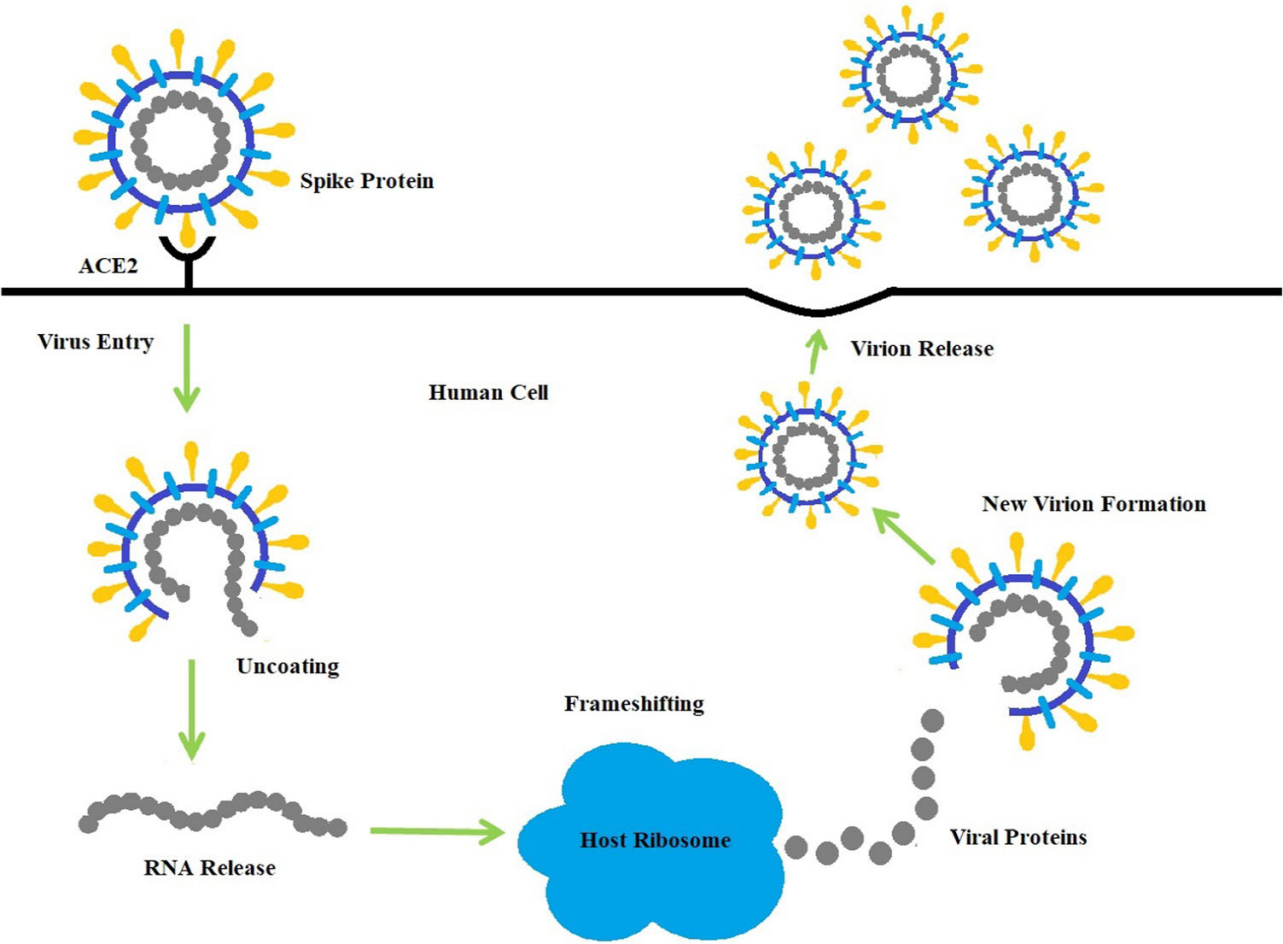
Coronavirus replication lifecycle

**Fig. 3:**
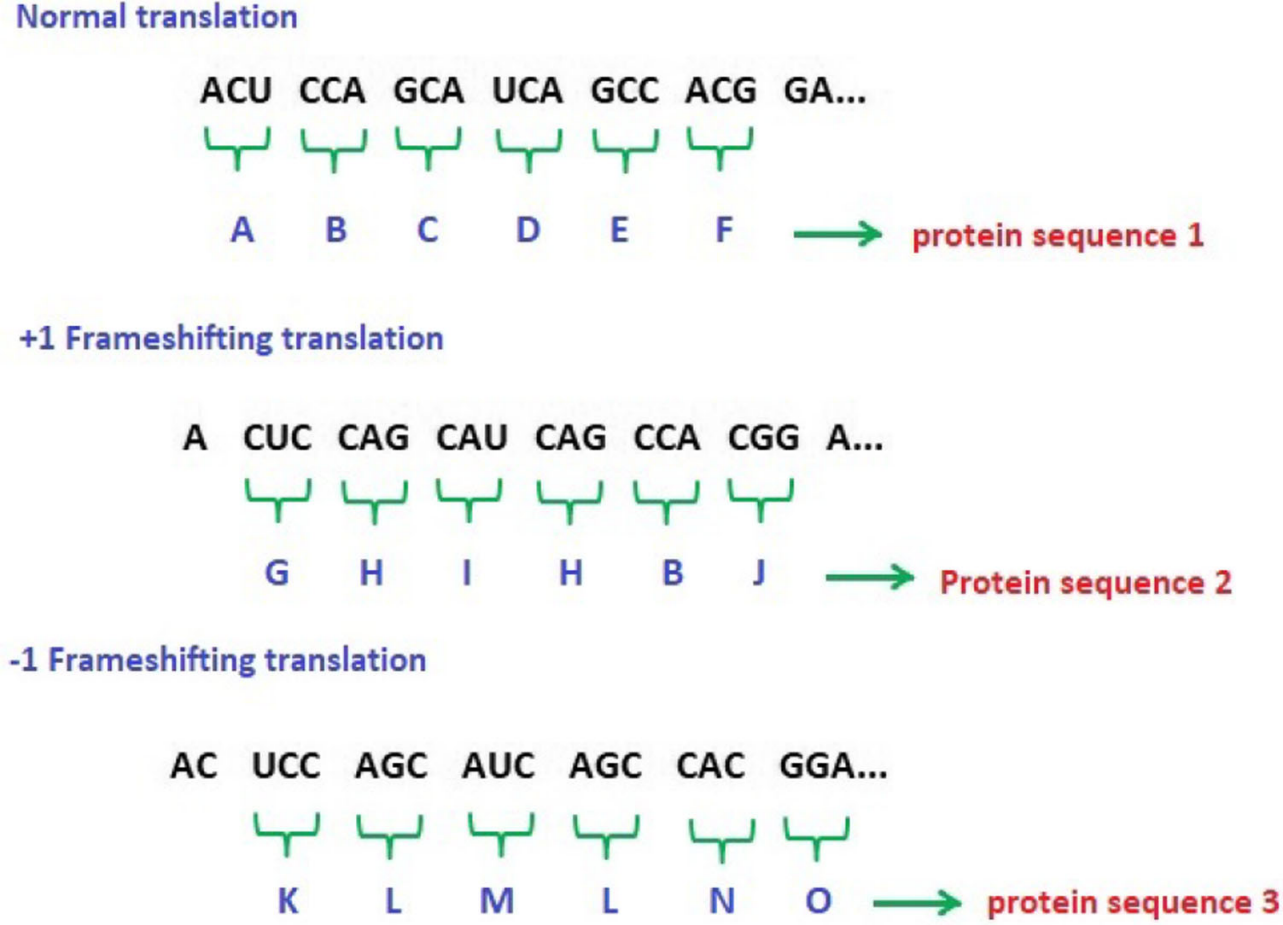
Frameshifting technique

COVIDOA is a recent evolutionary optimization algorithm inspired by the replication mechanism of Coronavirus when getting inside the human body [37]. The replication process of Coronavirus has four main stages as follows, see Fig. 2:

1. Virus entry and uncoating
  When a human is infected with COVID, the Coronavirus particles attach to the human cell via spike protein which is one of its structural proteins [39]. After getting inside the human cell, the virus contents are released.
2. Virus replication
  The virus tries to make more copies to hijack other human healthy cells. The virus's replication technique is called the frameshifting technique [38, 39]. Frameshifting is moving the reading frame of a protein sequence of the virus to another reading frame that leads to the creation of many new viral proteins that are then merged to form new virus particles. The frameshifting technique is presented in Fig. 3. As shown in the figure, in the replication process, the virus's mRNA (messenger Ribonucleic Acid) is translated into viral proteins by reading tri-nucleotides (e.g., ACU). Each tri-nucleotide is translated into single amino acid. Thus, shifting (backward or forward) the reading frame of the nucleotides sequence by any number (not divisible by 3) will create different sequences that will be translated into different viral proteins. According to this technique, the virus can create millions of new particles than will damage millions of human cells. There are many types of frameshifting techniques; however, the most popular is +1 frameshifting as follows [40]:
  • +1 frameshifting technique
  The elements of the parent virus particle (parent solution) are moved in the right direction by 1 step. As a result of +1 frameshifting, the first element is lost. In the proposed algorithm; the first element is set a random value in the range [Lb, Ub] as follows:
  10 $$ S_{k} \left( 1 \right) = {\text{rand}}\left( {{\text{Lb}},{\text{Ub}}} \right), $$
  11 $$ S_{k} \left( {2:D} \right) = P\left( {1:D - 1} \right), $$
  where *P* refers to the parent solution, $S_{k}$ is the *k*th generated viral protein, *D* is the problem dimension, and Lb and Ub are the lower and upper bounds for the variables in each solution.
3. Virus mutation
  Coronavirus uses the mutation technique to resist the human immune system [40]. In the proposed algorithm, the mutation is applied to the previously created new virus particle (solution) to produce a new one as follows:
  12 $$ Z_{i} = \left\{ {\begin{array}{*{20}l} r \hfill & {{\text{if rand}}\left( {0,1} \right) < {\text{MR}}} \hfill \\ {X_{i} } \hfill & {{\text{otherwise}}} \hfill \\ \end{array} } \right. $$
  where *X* is the solution before mutation, *Z* is the mutated solution, *X*_*i*_ and *Z*_*i*_ are the *i*^*th*^ element in the old and new solutions, respectively, *i* =1, …, D, and *r* is a random value in the range [Lb, Ub]. MR is the mutation rate.
4. New virion release
  The newly created virus particle leaves the infected cell targeting new healthy cells. In the proposed algorithm, if the fitness of the new solution is better than the parent solution fitness, the parent solution is replaced by the new one. Otherwise, the parent solution remains. The pseudocode of the COVID algorithm is as follows:

## COVIDOA with a chaotic map

In COVIDOA, each virus particle represents a solution in the population. The dimension of each solution is equal to the number of threshold values needed for segmentation plus 1. The first population solution is initialized randomly, where each element in the solution vector is assigned a value within the range of pixel intensities of the grayscale image. For the remaining solutions in the population, the initialization is done using chaotic maps to generate a uniformly distributed initial population [44, 45]. We used eight chaotic maps to enhance the quality of the initial population.

In the chaotic initialization, given the solution vector ${S}_{j.}$ The solution vector ${S}_{j+1}$ can be driven by the following formula:

1. Sine Chaotic map:
  13 $${S}_{j+1}=\frac{q}{4}\mathrm{sin}\left(\pi {S}_{j}\right), q=4$$
2. Singer Chaotic Map:
  14 $${S}_{j+1}=\beta \left(7.86{S}_{j}-23.31{{S}_{j}}^{2}+28.75{{S}_{j}}^{3}-13.302875{{S}_{j}}^{4}\right), \beta =1.07$$
3. Sinusoidal Chaotic Map:
  15 $${S}_{j+1}=u{{S}_{j}}^{2}\sin(\pi {S}_{j}), u=2.3$$
4. Chebyshev Chaotic Map:
  16 $${S}_{j+1}=\cos(\hbox{arccos}{S}_{j})$$
5. Tent Chaotic Map:
  17 $${S}_{j+1}=\left\{\begin{array}{ll}\frac{{S}_{j}}{0.7}&\quad {S}_{j}<0.7\\ \frac{10}{3}\left(1-{S}_{j}\right)&\quad {S}_{j}\ge 0.7\end{array}\right.$$
6. Logistic Chaotic Map:
  18 $${S}_{j+1}=u{S}_{j}\left(1-{S}_{j}\right),u=4$$
7. Iterative Chaotic Map:
  19 $${S}_{j+1}=sin\frac{u\pi }{{S}_{j}},u=0.7$$
8. Gauss/Mouse Chaotic Map:
  20 $${S}_{j+1}={e}^{-\alpha {{S}_{j}}^{2}}+\beta,\quad \alpha =4.90,\quad \beta =-0.58$$

Chaotic initialization is a modern technique used to ensure that the solutions of the initial population are uniformly distributed, which helps avoid the problem of getting stuck into local minima or maxima [46]. As discussed in the results section, we found that the Logistic chaotic map is the one that gives the best results.

## Results and discussion

In this section, we firstly provide a brief description of the datasets used for testing. Then, we show the parameter settings for the proposed and state-of-the-art algorithms. After that, the evaluation metrics used for comparing the results are explained in detail. Finally, we present the numerical results of running the proposed algorithm and its peers.

### Datasets

Six satellite images are selected from “NASA Visible Earth” [47] to prove the efficiency of the proposed algorithm in image segmentation. In addition to six benchmark images. These images have many variations, such as size and resolution. The test images and their histograms are shown in Table 1.

**Table 1:**
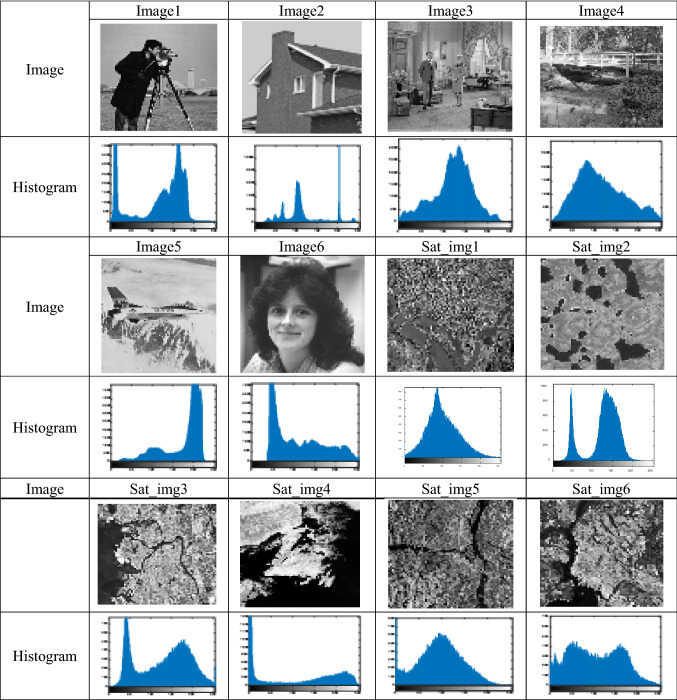
Test images and their histograms

### Parameter setting

The results of multilevel thresholding using the proposed algorithm are compared with eleven well-known metaheuristic algorithms. In comparison, we focused on the recently proposed algorithms to prove the superiority of the proposed algorithm. These algorithms are: Harris Hawks Optimization algorithm (HHO) [48], Reptile Search Algorithm (RSA) [49], Seagull Optimization algorithm (SOA) [50], Black Widow Optimization Algorithm (BWOA) [51], Marine Predators Algorithm (MPA) [52], Aquila optimizer (AO) [53], Slime Mold Algorithm (SMA) [54], Arithmetic Optimization Algorithm (AOA) [55], Jellyfish Optimization algorithm (JOA) [56], Moth–flame optimization algorithm (MFO) [57], Sine Cosine Algorithm (SCA) [58]. The reasons for selecting these algorithms for comparison are as follows:

- They have proved their superior performance in optimization problems, especially image segmentation.
- Most of them are recent and published in reputable sources.
- Their MATLAB implementations are publicly available on the MATLAB website (https://www.mathworks.com/).

The parameters of all algorithms are set as mentioned in their original papers. In all algorithms, the population size is 50, and the maximum number of iterations to 100. All algorithms were run 20 times, and the best-obtained results are reported in the results section.

### Performance metrics

The performance of the proposed algorithm is evaluated using several performance metrics, including Mean Square Error (MSE), peak signal-to-noise ratio (PSNR), structural similarity index (SSIM), Feature Similarity Index (FSIM), Normalized Correlation Coefficient (NCC), and best fitness in addition to the Wilcoxon rank-sum test.

PSNR, SSIM, and NCC are used to measure the quality of the segmented images, while best fitness is measured to prove the ability of the proposed algorithm to find optimum solutions, and the Wilcoxon rank-sum test is utilized to prove the statistical significance of the proposed algorithm as follows:

- Best fitness
  The maximum fitness is obtained from running the proposed ad state-of-the-art algorithms with the proposed hybrid fitness function equations (9). By trial and error approach, we found that the proposed algorithm yields better results at a = 0.5 and b = 0.5.
- Mean Square Error (MSE)
  MSE is commonly used to estimate the error between the original and segmented images. It can be calculated as follows:
  21 $$\hbox{MSE}=\frac{1}{M\times N}\sum_{i=1}^{M}\sum_{j=1}^{N}{\left[F\left(i,j\right)-f(i,j)\right]}^{2}$$
  *F*(*i*, *j*) is the original image*, f*(*i*, *j*) is the segmented image, and $M\times N$ refers to the image size.
- Peak signal-to-noise ratio (PSNR)
  PSNR is commonly used to quantify the quality of images. It refers to the ratio between the segmented image power and noise power
  22 $$\hbox{PSNR}=10 \hbox{Log}_{10}\left(\frac{{255}^{2}}{MSE}\right)$$
- Structural similarity index (SSIM)
  SSIM is used to quantify the structural similarity between the original and segmented images as follows:
  23 $$\hbox{SSIM}(F,f)=\frac{(2{\mu }_{F}{\mu }_{f}+{C}_{1})(2{\sigma }_{Ff}+{C}_{2})}{({\mu }_{F}^{2}{\mu }_{f}^{2}+{C}_{1})({\sigma }_{F}^{2}{\sigma }_{f}^{2}+{C}_{2})}$$
  where *F* and *f* are the original and segmented images. ${\mu }_{F}$ and ${\mu }_{f}$ are the mean intensity of *F* and *f,* respectively. ${\sigma }_{F}^{2}$ and ${\sigma }_{f}^{2}$ are the variance of *F* and *f,* respectively. *C*_1_ = 6.502 and *C*_2_ = 58.522.
- Feature similarity index (FSIM)
  FSIM is used to measure the similarity in the structure of the two images as follows:
  24 $$\hbox{FSIM}\left(F,f\right)=\frac{{\sum }_{x\in\Omega }{S}_{L}\left(x\right)\cdot\hbox{PC}_{m}(x)}{{\sum }_{x\in\Omega }\hbox{PC}_{m}(x)}$$
  where ${\mathrm{S}}_{\mathrm{L}}\left(\mathrm{x}\right)$ refers to the similarity between the two images, *PC* is the phase congruence, and $\Omega$ refers to the spatial domain of the image. The maximum value of the FSIM that corresponds to complete similarity is 1.
- Normalized correlation coefficient (NCC)
  NCC is used to measure the extent to which two images are related. The absolute value of NCC ranges from 0 to 1, where 0 indicates that the two images have no relation and 1 indicates the strongest possible relation. The higher the absolute value of NCC, the stronger the relationship between the two images. NCC between the original and segmented images *F*(*i, j*) and *f*(*i*, *j*) is calculated as follows:
  25 $$\hbox{NCC}=\frac{\sum_{i=0}^{M-1}\sum_{j=0}^{N-1}(F(i,i)\times f(i,j))}{\sqrt{\sum_{i=0}^{M-1}\sum_{j=0}^{N-1}(F(i,i)\times F(i,j))\times \sum_{i=0}^{M-1}\sum_{j=0}^{N-1}(f(i,i)\times f(i,j))}}$$
- Wilcoxon rank-sum test
  The Wilcoxon rank-sum test is a nonparametric statistical test used to measure the statistical difference between two related methods [59]. We conducted the Wilcoxon rank-sum test with a 5% significance level to prove the proposed algorithm's statistical significance compared to the other algorithms.

### Experimental results

This section presents the numerical results of running the proposed algorithm to select the optimum threshold values using the proposed hybrid fitness function with chaotic initialization. These results are compared with the state-of-the-art algorithms in best fitness, MSE, PSNR, SSIM, FSIM, NCC, and Wilcoxon rank-sum test. The experiments have been performed using 6, 10, 14, 18, 22, and 26 thresholds.

Firstly, a comparison between the results of the various chaotic maps is conducted to demonstrate that the logistic map gives the best results among the others, as shown in Table 2, where k represents the number of threshold values. The results in the table are calculated by taking the average value for each criterion for all the images in the two mentioned datasets. It is obvious from the table that using chaotic maps increases the diversity of the solutions and yields better results.

**Table 2:** The results obtained from using different chaotic maps in the initialization phase of COVIDOA

Chaotic map	*K*	MSE	PSNR	SSIM	FSIM	NCC	Fitness
No map	6	168.8419	21.4969	0.6504	0.8924	0.9782	1894.9935
	10	106.5881	27.0012	0.6890	0.9566	0.9834	1928.7658
	14	84.7620	28.5479	0.7705	0.9642	0.9935	1941.5097
	18	60.7663	28.7492	0.7875	0.9722	0.9912	1945.4065
	22	52.8740	30.6873	0.8045	0.9805	0.9928	1953.8932
	26	30.1434	32.6737	0.8799	0.9810	0.9972	1958.2405
Sine	6	168.5419	22.1969	0.6604	0.8924	0.9852	1895.0965
	10	106.2856	27.0572	0.7559	0.9587	0.9959	1929.3268
	14	82.5727	28.7403	0.7758	0.9672	0.9972	1941.5097
	18	58.0161	30.2405	0.7973	0.9791	0.9982	1949.4645
	22	50.9747	31.0563	0.8129	0.9815	0.9984	1955.3632
	26	29.8427	33.3797	0.8893	0.9819	0.9987	1959.4009
Singer	6	151.2242	23.1725	0.6781	0.9084	0.9874	1897.9444
	10	101.0654	26.7873	0.7408	0.9486	0.9952	1928.789
	14	87.9834	28.3421	0.7717	0.9684	0.9971	1940.9694
	18	65.3103	29.8663	0.7918	0.9766	0.9980	1949.2349
	22	55.7129	30.6173	0.8007	0.9809	0.9984	1955.1724
	26	29.2908	33.4635	0.9035	0.9817	0.9987	1959.7431
Sinusoidal	6	153.5743	23.4933	0.6871	0.9163	0.9893	1899.6754
	10	102.7959	27.3056	0.7541	0.9580	0.9961	1931.157
	14	89.7622	28.3461	0.7706	0.9675	0.9970	1941.1573
	18	61.2596	30.1179	0.7952	0.9773	0.9982	1948.9674
	22	48.5882	31.1851	0.8137	0.9835	0.9986	1955.0124
	26	26.5781	33.8854	0.9086	0.9829	0.9988	1960.2382
Chebyshev	6	152.2352	23.3680	0.6861	0.9132	0.9884	1899.5238
	10	105.2388	26.5905	0.7511	0.9511	0.9947	1929.4676
	14	78.5240	28.9274	0.7760	0.9706	0.9975	1941.7624
	18	65.4571	29.9182	0.7971	0.9776	0.9981	1949.1023
	22	50.1469	31.1088	0.8109	0.9822	0.9986	1955.387
	26	44.1702	31.6593	0.8181	0.9851	0.9987	1960.009
Tent	6	151.6123	22.4326	0.6678	0.9032	0.9835	1896.6805
	10	105.7787	26.9881	0.7495	0.9575	0.9957	1929.9373
	14	85.7759	28.5388	0.7738	0.9720	0.9974	1941.6714
	18	63.0602	30.0181	0.7932	0.9780	0.9982	1949.1906
	22	52.6716	30.8763	0.8067	0.9824	0.9986	1955.206
	26	26.8906	33.8348	0.9207	0.9846	0.9988	1960.4115
Logistic	6	153.1303	23.4859	0.6864	0.9180	0.9891	1899.3585
	10	103.7412	27.1904	0.7520	0.9580	0.9960	1930.1119
	14	74.1476	29.1405	0.7818	0.9742	0.9978	1942.1868
	18	59.4670	30.2672	0.7981	0.9795	0.9983	1949.7015
	22	51.2212	30.9980	0.8087	0.9833	0.9986	1955.3627
	26	42.8955	31.7859	0.8208	0.9854	0.9988	1959.9374
Iterative	6	166.2826	22.2247	0.6631	0.8945	0.9847	1895.9168
	10	111.5121	26.9058	0.7519	0.9586	0.9959	1929.6884
	14	74.6758	28.9529	0.7859	0.9713	0.9973	1941.4396
	18	60.6064	30.2592	0.7953	0.9779	0.9983	1949.5098
	22	51.4856	31.0078	0.8123	0.9808	0.9984	1954.9514
	26	44.7721	31.6008	0.8176	0.9845	0.9987	1960.1318
Gaussian	6	150.6483	23.7096	0.6915	0.9160	0.9899	1899.3918
	10	117.9716	26.3555	0.7336	0.9523	0.9948	1929.4217
	14	79.1685	28.5785	0.7713	0.9706	0.9971	1941.2494
	18	61.5068	29.8459	0.7900	0.9755	0.9981	1948.758
	22	53.2253	30.8262	0.8035	0.9814	0.9985	1954.8612
	26	23.6472	34.3930	0.9258	0.9258	0.9864	0.9990

The higher PSNR, SSIM, FSIM, NCC, and fitness values and lower MSE values resulting from the chaotic logistic map demonstrate its robustness. Hence, the chaotic logistic map is utilized while performing further experiments.

Table 3 proves that the hybrid fitness function is more robust than using the Otsu or Kapur methods separately. It is clear from the table that the quality of the segmented images using COVIDOA with the hybrid fitness function is higher than Otsu and Kapur methods according to MSE, PSNR, SSIM, FSIM, and NCC values.

**Table 3:** Comparison between the performance of Otsu, Kapur, and hybrid fitness function

Fitness function	*K*	MSE	PSNR	SSIM	FSIM	NCC
Otsu	6	153.7986	23.2120	0.6822	0.9143	0.9877
	10	106.6566	26.8796	0.7419	0.9573	0.9955
	14	**73.2163**	29.1834	0.7821	0.9729	0.9977
	18	60.5699	30.2128	0.7971	0.9784	0.9982
	22	47.8161	31.1670	0.8098	0.9834	0.9986
	26	29.2131	33.4745	0.8753	0.9835	0.9987
Kapur	6	165.0680	22.6135	0.6831	0.9048	0.9870
	10	166.7912	23.1803	0.7316	0.9319	0.9902
	14	118.5607	26.7798	0.7461	0.9512	0.9954
	18	89.7075	27.8912	0.7638	0.9564	0.9956
	22	55.0984	30.7032	0.8051	0.9796	0.9984
	26	54.2876	30.7673	0.8069	0.9792	0.9982
Hybrid	6	**153.1303**	**23.4859**	**0.6864**	**0.9180**	**0.9891**
	10	**103.7412**	**27.1904**	**0.7520**	**0.9580**	**0.9960**
	14	**73.1476**	**29.1405**	**0.7818**	**0.9742**	**0.9978**
	18	**59.4670**	**30.2672**	**0.7981**	**0.9795**	**0.9983**
	22	**47.2212**	**30.9980**	**0.8087**	**0.9838**	**0.9986**
	26	**25.6045**	**33.7859**	**0.8608**	**0.9854**	**0.9988**

All algorithms have been applied to solve multilevel thresholding problems for both the standard and satellite images to show the effectiveness of the proposed algorithm against other proposed methods. The results for the six benchmark images are shown in Tables 4, 5, 6, 7, 8 and 9 for fitness, MSE, PSNR, SSIM, FSIM, and NCC, respectively. In contrast, the results for the six satellite images are shown in Tables 10, 11, 12, 13, 14 and 15. The values in these tables, highlighted in bold, indicate the best results.

**Table 4:** The fitness results of benchmark image segmentation using hybrid fitness function for all algorithms

Image	*K*	RSA [47]	SOA [48]	BWOA [49]	MPA [50]	AO [51]	SMA [52]	AOA [53]	JOA [54]	MFO [55]	HHO [46]	SCA [56]	Proposed COVID
Image1	6	1872.4	1878.7	1881.4	1896.1	1894.8	1899.6	1896.3	1899.6	1899.6	1899.7	1883.5	**1899.8**
	10	1915.7	1925.2	1919.4	1915.3	1929.6	1931.1	1922.8	1929.4	1930.8	1931.0	1900.1	**1931.2**
	14	1924.6	1927.7	1935.0	1938.7	1939.7	1941.6	1931.6	1940.6	1942.0	1940.2	1921.8	**1942.1**
	18	1940.3	1946.8	1944.3	1948.1	1949.3	1950.1	1941.1	1948.3	**1950.2**	1950.2	1941.3	1949.9
	22	1950.4	1952.7	1950.6	1954.1	1953.9	1955.4	1949.2	1953.9	1956.1	1956.1	1950.3	**1958.9**
	26	1953.3	1957.2	1955.0	1959.7	1959.4	1959.6	1950.2	1958.9	1959.9	**1960.9**	1953.2	1960.5
Image2	6	1616.7	1628.0	1637.2	1651.5	1653.4	**1656.7**	1653.2	1656.2	1656.6	**1656.7**	1652.2	**1656.7**
	10	1671.2	1678.0	1674.6	1674.9	1679.7	1680.4	1673.7	1677.9	1680.5	1680.4	1670.7	**1680.6**
	14	1684.5	1686.8	1684.0	1689.8	1688.5	**1690.9**	1687.4	1689.7	1690.7	1690.0	1683.0	**1690.9**
	18	1691.1	1691.2	1692.3	1697.9	1695.1	1697.4	1688.8	1696.7	1697.8	1695.8	1691.3	**1698.0**
	22	1696.0	1698.8	1698.3	1700.6	1702.1	1702.5	1695.1	1700.9	1702.9	1701.7	1695.7	**1703.1**
	26	1705.5	1704.5	1702.5	1703.6	1703.1	1707.2	1698.8	1706.8	1707.0	1707.3	1699.1	**1707.7**
Image3	6	941.15	944.71	948.23	950.34	950.00	950.43	947.88	950.12	**950.45**	949.21	945.85	950.34
	10	977.67	979.66	978.22	984.81	988.45	989.27	981.34	985.53	986.70	988.45	985.64	**989.67**
	14	991.60	995.73	995.45	1001.7	1001.3	**1002.0**	993.02	1001.4	1001.3	1001.3	998.56	1001.8
	18	999.35	1002.7	1001.0	**1010.9**	1008.8	1009.3	1000.8	1008.4	1010.1	1008.0	1001.5	1010.2
	22	1009.0	1009.2	1012.6	1018.1	1017.5	1016.8	1008.9	1009.45	1010.2	1013.5	1010	**1018.9**
	26	1011.4	1017.0	1018.7	1020.0	1018.5	1020.1	1011.7	1011.2	1011.43	1022.0	1011.3	**1023.5**
Image4	6	1408.8	1431.6	1405.3	**1433.0**	1432.0	1432.9	1425.0	1432.8	1432.9	**1433.0**	1428.1	1432.7
	10	1448.9	1443.4	1455.5	1475.8	1472.8	1475.8	1464.7	1474.4	1475.9	1475.9	1455.7	1478.7
	14	1490.5	1486.5	1481.6	1493.7	1491.0	1491.5	1478.3	1491.7	1492.3	1491.3	1485.2	**1492.5**
	18	1491.5	1496.3	1493.6	1499.5	1498.1	1500.1	1492.2	1499.6	1501.0	1498.9	1485.7	**1501.1**
	22	1501.5	1499.5	1500.8	1507.8	1504.2	1507.9	1494.7	1507.5	1506.9	1507.8	1497.0	**1508.1**
	26	1502.9	1505.4	1505.5	1513.2	1508.8	1512.9	1504.0	1511.9	1512.4	1511.6	1505.9	**1513.3**
Image5	6	1033.0	1032.3	1050.1	1050.2	1054.3	1051.7	1052.61	1048.35	1046.20	1054.2	1049.6	**1054.4**
	10	1052.0	1051.8	1052.6	1052.4	1057.2	1054.1	1056.4	1052.2	**1068.9**	1065.5	1052.5	1057.6
	14	1078.9	1082.8	1087.2	1090.3	1086.8	1086.0	1080.2	1078.4	1081.3	**1088.2**	1083.4	**1088.2**
	18	1088.9	1091.2	1090.0	1096.5	1092.3	1089.3	1088.3	1081.8	1086.4	1095.6	1085.3	**1096.7**
	22	1095.4	1098.7	1095.3	1099.0	1094.3	1094.4	1092.6	1087.3	1090.3	**1100.3**	1088.0	1100.2
	26	1099.3	1100.2	1099.2	1102.6	1100.5	1095.3	1096.2	1093.4	1095.6	**1103.4**	1093.2	1103.2
Image6	6	1855.7	1872.8	1873.1	1873.8	1873.0	1871.1	1878.4	1837.8	1846.7	1873.5	1863.4	**1878.5**
	10	1882.1	1893.3	1903.6	1903.9	1905.3	1893.8	1878.7	1899.0	1894.5	**1906.1**	1880.4	**1906.1**
	14	1895.9	1916.2	1916.8	**1919.8**	1916.9	1909.2	1901.2	1910.0	1907.8	1919.2	1899.1	1918.2
	18	1905.6	1922.5	1922.3	1926.5	1917.3	1920.3	1908.8	1924.7	1913.8	1927.5	1890.0	**1928.6**
	22	1917.3	1928.5	1926.8	1928.8	1918.4	1927.8	1920.6	1928.9	1915.3	1931.9	1906.7	**1932.5**
	26	1923.7	1928.4	1929.8	1931.0	1919.2	1931.1	1924.4	1931.2	1919.0	**1935.1**	1912.8	1934.5
Average		1506.2	1511.2	1511.6	1517.0	1515.9	1516.8	1510.2	1514.3	1514.7	1518.6	1507.1	**1519.4**

**Table 5:** The MSE results of benchmark image segmentation using hybrid fitness function for all algorithms

Image	*K*	Algorithm											
		RSA	SOA	BWOA	MPA	AO	SMA	AOA	JOA	MFO	HHO	SCA	Proposed COVID
Image1	6	154.23	159.45	165.34	**147.34**	152.65	153.65	176.45	157.76	155.25	156.98	169.45	148.41
	10	133.10	112.85	136.24	99.51	**102.81**	109.12	140.15	108.31	105.86	108.74	142.65	104.23
	14	110.65	88.34	89.65	78.65	80.47	85.37	122.76	86.34	79.34	77.45	103.67	**76.34**
	18	88.94	56.84	72.23	68.87	49.58	49.93	90.47	67.11	41.28	50.56	85.07	**48.30**
	22	67.34	43.54	65.75	58.65	45.65	34.67	76.45	53.34	39.45	43.67	67.34	**32.56**
	26	58.50	30.39	56.99	30.77	40.69	23.93	55.32	31.49	28.05	**21.84**	30.88	23.56
Image2	6	233.54	248.65	244.63	259.65	242.54	234.76	253.65	255.46	230.54	216.91	254.64	**215.10**
	10	139.96	165.35	161.12	160.41	138.22	143.53	174.94	170.24	142.36	135.71	168.90	**117.44**
	14	61.76	101.10	71.69	59.34	80.60	73.48	76.23	60.81	61.54	68.55	112.43	**57.18**
	18	58.23	64.63	49.64	55.74	39.65	38.65	70.45	52.65	46.34	70.05	62.63	**29.85**
	22	55.79	40.84	42.59	44.53	37.95	31.01	66.22	42.48	31.91	36.30	36.48	**27.95**
	26	63.15	36.52	63.19	26.98	32.29	19.66	37.82	23.45	25.61	24.00	25.87	**17.06**
Image3	6	176.86	175.07	177.91	169.00	171.53	169.22	**161.47**	170.55	167.91	171.99	163.58	167.43
	10	105.34	123.56	127.65	112.56	106.35	104.64	129.45	184.35	113.83	114.24	143.54	**101.34**
	14	90.46	90.97	83.24	71.47	80.23	67.93	96.38	177.78	87.45	72.79	112.54	**64.17**
	18	81.59	82.33	77.01	43.87	59.45	46.06	94.01	137.46	44.02	61.36	72.82	**43.59**
	22	73.53	59.75	47.53	41.46	44.23	39.45	72.45	125.45	40.34	39.34	86.3533	**35.34**
	26	59.81	40.86	37.45	33.22	36.90	25.34	43.93	118.31	28.42	25.67	64.68	**24.30**
Image4	6	181.30	184.23	189.09	181.67	180.97	182.04	182.96	180.90	181.79	181.93	183.11	**180.00**
	10	154.65	152.65	153.65	137.45	154.76	142.65	138.65	122.90	124.64	125.36	147.345	**122.65**
	14	122.54	105.34	116.34	114.45	110.24	100.34	102.53	86.99	**82.92**	84.16	120.34	86.36
	18	86.96	72.55	74.22	71.99	67.15	65.08	85.00	66.28	59.57	67.41	99.34	**55.99**
	22	62.30	65.22	70.01	42.40	57.19	39.24	86.16	40.11	42.27	40.90	78.34	**38.22**
	26	58.44	49.26	51.27	31.71	44.47	28.07	45.56	33.56	30.97	32.21	51.05	**26.23**
Image5	6	141.90	157.28	169.74	152.86	153.74	150.53	152.37	206.52	239.85	153.04	165.23	**115.91**
	10	99.34	110.43	114.24	94.23	89.34	122.54	102.34	144.31	225.34	90.43	142.45	**85.32**
	14	80.43	82.43	65.34	45.71	59.65	80.34	77.52	123.34	213.06	57.61	117.71	**55.11**
	18	71.42	68.72	50.26	40.53	48.53	67.87	64.24	104.31	195.23	45.34	96.34	**43.54**
	22	57.76	44.35	39.76	38.43	42.53	53.23	51.34	81.35	168.43	39.33	82.53	**38.23**
	26	41.98	29.65	27.13	**25.26**	34.22	42.45	40.59	82.92	147.73	27.37	66.05	27.96
Image6	6	**198.71**	195.46	199.33	201.92	192.74	205.87	199.33	213.90	221.96	220.35	234.875	210.34
	10	176.45	165.34	149.93	154.23	173.75	168.76	177.44	176.54	198.76	143.09	176.26	**140.07**
	14	134.35	116.33	97.72	104.25	125.30	123.26	131.73	125.50	170.02	102.47	115.16	**83.40**
	18	103.65	92.67	88.65	83.72	92.65	99.54	105.45	95.3422	132.43	78.46	89.34	**55.34**
	22	66.34	64.56	84.64	48.23	90.34	85.36	95.23	69.2345	103.46	48.23	82.65	**45.34**
	26	43.57	40.24	51.97	25.73	87.74	58.76	64.53	55.0519	82.38	26.72	80.98	**23.50**
Average		102.63	97.71	98.97	87.68	92.97	90.73	106.71	112.01	113.61	85.01	112.01	**76.87**

**Table 6:** The PSNR results of benchmark image segmentation using hybrid fitness function for all algorithms

Image	*K*	Algorithm											
		RSA	SOA	BWOA	MPA	AO	SMA	AOA	JOA	MFO	HHO	SCA	Proposed COVID
Image1	6	20.64	21.54	21.53	23.54	23.11	23.54	21.56	22.56	23.14	23.22	24.68	**27.71**
	10	24.12	25.96	25.03	27.15	27.00	27.08	25.53	26.92	27.21	27.06	25.98	**28.23**
	14	26.32	29.16	27.54	28.64	29.45	29.67	26.45	27.53	28.89	28.45	26.87	**29.74**
	18	28.04	30.29	29.09	29.33	31.29	**32.03**	28.19	30.79	31.91	31.88	28.31	30.94
	22	29.54	31.43	30.12	30.67	30.01	**33.53**	29.54	31.53	32.53	32.53	30.53	32.84
	26	30.16	33.15	30.49	33.24	32.03	34.50	30.57	32.98	33.64	34.73	33.01	**34.80**
Image2	6	20.21	20.57	20.42	20.78	21.35	20.454	18.45	20.17	**20.96**	20.72	19.53	20.95
	10	24.24	24.71	24.74	25.02	25.82	24.95	22.95	24.76	25.65	25.76	24.00	**26.37**
	14	28.55	27.54	28.24	30.10	28.50	29.27	28.84	29.97	29.88	29.46	26.51	**30.25**
	18	29.56	29.45	28.56	31.02	30.53	31.46	28.22	30.76	31.56	29.60	29.56	**33.26**
	22	30.18	31.09	28.81	31.52	32.09	33.21	29.49	31.79	33.00	32.40	26.73	**33.62**
	26	27.42	32.36	30.04	33.76	32.66	35.17	31.41	34.42	33.99	34.32	30.00	**35.80**
Image3	6	21.67	21.82	21.13	22.22	22.14	22.20	20.88	20.16	22.31	22.09	22.14	**22.30**
	10	25.54	25.55	24.89	25.88	25.43	25.75	23.63	22.56	26.69	26.58	23.29	**26.78**
	14	27.83	27.77	28.21	29.49	29.55	29.65	27.01	23.13	29.77	29.37	26.44	**29.78**
	18	27.88	28.73	29.12	**31.73**	30.35	31.42	27.78	24.41	31.65	30.20	28.56	31.68
	22	28.45	30.54	30.89	32.23	31.34	32.67	28.94	25.77	32.68	32.78	29.21	**33.22**
	26	29.91	31.99	32.39	32.91	32.42	33.15	30.99	28.37	33.95	34.03	29.98	**34.27**
Image4	6	19.69	20.48	19.86	20.48	20.61	20.55	20.46	20.56	20.53	20.45	20.12	**20.63**
	10	22.71	23.67	22.78	23.62	23.89	**24.94**	22.54	24.63	24.55	24.34	22.84	24.64
	14	24.78	25.39	27.45	26.39	26.97	27.10	26.43	28.42	28.38	**28.55**	25.43	28.38
	18	27.66	28.65	30.30	29.55	29.46	29.98	28.14	29.86	29.84	29.78	27.91	**30.52**
	22	29.86	29.77	29.29	31.85	30.54	32.19	27.89	32.07	31.81	32.01	28.22	**32.20**
	26	30.05	30.92	29.45	33.11	31.63	33.64	31.39	32.87	33.22	33.04	30.10	**33.92**
Image5	6	23.69	23.05	20.27	23.12	23.02	23.53	23.11	19.89	13.17	23.15	22.54	**24.12**
	10	25.44	25.68	20.87	27.33	26.49	26.37	25.78	20.56	16.97	27.40	23.01	**27.45**
	14	27.54	27.80	21.14	**30.88**	29.51	28.34	27.02	23.25	18.54	30.20	23.56	29.84
	18	29.16	30.60	21.23	31.67	30.34	30.15	29.01	25.98	20.44	31.66	25.76	**31.87**
	22	30.43	31.44	21.54	32.71	31.87	31.57	30.37	27.41	21.76	32.44	28.21	**32.75**
	26	31.78	32.80	22.47	34.10	32.65	32.43	31.40	28.05	23.09	**33.75**	29.57	33.57
Image6	6	16.74	17.44	17.20	17.16	17.48	17.69	17.37	18.44	18.04	19.14	14.24	**19.32**
	10	18.34	18.81	20.04	19.43	8.78	18.44	19.21	20.22	18.71	20.45	15.40	**20.67**
	14	21.08	20.21	22.83	21.33	19.88	19.78	20.92	21.85	19.48	22.76	22.79	**25.09**
	18	25.85	25.31	23.42	25.97	22.07	22.63	24.84	24.60	22.65	25.98	23.85	**26.68**
	22	27.71	27.22	25.11	27.64	24.62	24.57	26.89	27.45	25.34	27.76	25.24	**27.89**
	26	31.60	31.83	25.99	30.29	27.16	28.89	29.92	29.11	28.26	**32.75**	27.74	31.97
Average		26.23	27.07	25.34	27.94	27.00	27.84	26.19	26.21	26.22	28.35	25.60	**29.00**

**Table 7:** The SSIM results of benchmark image segmentation using hybrid fitness function for all algorithms

Image	*K*	Algorithm											
		RSA	SOA	BWOA	MPA	AO	SMA	AOA	JOA	MFO	HHO	SCA	Proposed COVID
Image1	6	0.6478	0.6679	0.6437	0.6856	0.6740	0.6902	0.6478	0.6511	0.6547	0.6876	0.6805	**0.6934**
	10	0.7161	**0.8293**	0.7198	0.7580	0.7505	0.7612	0.7329	0.7524	0.7592	0.7574	0.7167	0.7596
	14	0.7432	0.8422	0.8156	0.7710	0.8239	0.8349	0.7783	0.8490	0.8657	0.8783	0.7750	**0.8867**
	18	0.7713	0.8590	0.8557	0.7864	0.8720	0.9110	0.7924	0.8976	0.9025	0.9208	0.7971	**0.9294**
	22	0.8002	0.8867	0.9053	0.8809	0.8890	0.9255	0.8089	0.9278	0.9100	0.9330	0.9217	**0.9321**
	26	0.8152	0.9195	0.92278	0.9322	0.8936	**0.9438**	0.8224	0.9368	0.9296	0.9501	0.9321	0.9412
Image2	6	0.9790	0.8021	0.8066	0.7989	0.8067	0.8110	0.7867	0.7726	0.8092	**0.8122**	0.7879	0.8119
	10	0.8616	0.8737	0.8765	0.8638	0.8690	**0.8975**	0.8738	0.8567	0.8941	0.8602	0.8778	**0.8975**
	14	0.8878	0.9118	0.9310	0.9168	0.9368	0.9302	0.9045	0.9186	0.9341	0.9299	0.9011	**0.9446**
	18	0.8978	0.9255	0.9322	0.9243	0.9421	0.9438	0.9214	0.9276	0.9435	0.9430	0.9092	**0.9559**
	22	0.9037	0.9461	0.9354	0.9319	0.9526	0.9570	0.9376	0.9490	0.9508	0.9440	0.9201	**0.9651**
	26	0.9387	0.9537	0.9383	0.9569	0.9495	0.9671	0.9197	0.9585	0.9678	0.9537	0.9231	**0.9708**
Image3	6	0.7421	0.7524	0.7359	0.7605	0.7600	0.7606	0.7148	0.7572	0.7619	0.7589	**0.7659**	0.7604
	10	0.8043	0.8122	0.8155	0.8623	0.8381	0.8477	0.7834	0.7790	0.8719	0.8761	0.8452	**0.8811**
	14	0.8815	0.8882	0.8959	0.9167	0.9032	0.9195	0.8744	0.7909	0.9177	0.9168	0.8977	**0.9178**
	18	0.8953	0.9022	0.9104	**0.9445**	0.9231	0.9421	0.8906	0.8029	0.9395	0.9293	0.9040	0.9404
	22	0.9149	0.9236	0.9378	0.9448	0.9422	0.9544	0.9108	0.8111	0.8546	0.9521	0.9100	**0.9548**
	26	0.9224	0.9367	0.9453	0.9538	0.9505	0.9648	0.9271	0.8380	0.8695	0.9628	0.9223	**0.9650**
Image4	6	0.7817	0.7466	0.7565	0.7468	0.7483	0.7496	0.7459	0.7468	**0.7508**	0.7472	0.7277	0.7497
	10	0.8548	0.8773	0.8256	0.8534	0.8611	0.8802	0.8624	0.9107	0.9117	0.9120	0.8517	**0.9212**
	14	0.8978	0.9182	0.9089	0.9128	0.9048	0.9367	0.8948	0.9319	0.9356	0.9359	0.9115	**0.9369**
	18	0.9154	0.9366	0.9315	0.9483	0.9399	0.9581	0.9210	0.9512	0.9459	0.9512	0.9241	**0.9544**
	22	0.9433	0.9445	0.9404	0.9663	0.9558	0.9682	0.9212	0.9652	0.9665	0.9686	0.9376	**0.9694**
	26	0.9482	0.9570	0.9356	0.9743	0.9623	0.9760	0.9571	0.9724	0.9734	0.9738	0.9520	**0.9762**
Image5	6	**0.8200**	0.8149	0.7229	0.8117	0.8101	0.8102	0.8174	0.7622	0.7187	0.8123	0.8185	**0.8200**
	10	0.9005	0.8945	0.7423	0.9012	0.8898	0.8873	0.8789	0.7790	0.8217	0.9012	0.8271	**0.9103**
	14	0.9178	0.8898	0.7656	**0.9336**	0.9241	0.8980	0.8802	0.7909	0.8289	0.9218	0.8576	0.9284
	18	0.9306	0.9168	0.7847	**0.9390**	0.9285	0.9143	0.9155	0.8867	0.83	0.9356	0.9231	0.9348
	22	0.9403	0.9255	0.9143	0.9450	0.9377	0.9252	0.9214	0.9045	0.8442	0.9453	0.9401	**0.9520**
	26	0.9454	0.9435	0.9226	0.9540	0.9480	0.9410	0.9370	0.9114	0.8513	0.9507	0.9460	**0.9583**
Image6	6	0.4321	0.4556	0.4516	0.4510	0.4603	**0.6710**	0.4746	0.6245	0.6288	0.6028	0.5786	0.6391
	10	0.5514	0.5329	0.5901	0.5484	0.5021	0.6793	0.6088	0.6571	0.6664	0.6149	0.6176	**0.6985**
	14	0.6518	0.6706	0.7308	0.6720	0.6006	0.7921	0.6500	0.7375	0.6892	0.6868	0.7676	**0.7927**
	18	0.8955	0.8562	0.7634	0.7065	0.7108	0.8875	0.8141	0.8522	0.8210	0.9075	0.8018	**0.9088**
	22	0.9120	0.9014	0.7890	0.8840	0.8078	0.9101	0.8572	0.9005	0.8670	**0.9275**	0.8378	0.9200
	26	0.9178	0.9289	0.8234	0.9312	0.8789	0.9120	0.8946	0.9115	0.8818	0.9378	0.8673	**0.9380**
Average		0.8411	0.8539	0.8284	0.8519	0.8457	0.8794	0.8327	0.8436	0.8519	0.8777	0.8465	**0.8893**

**Table 8:** The FSIM results of benchmark image segmentation using hybrid fitness function for all algorithms

Image	*K*	Algorithm											
		RSA	SOA	BWOA	MPA	AO	SMA	AOA	JOA	MFO	HHO	SCA	Proposed COVID
Image1	6	0.8859	0.9055	0.8953	0.9177	0.9145	0.9121	0.8982	0.9102	**0.9186**	0.9111	0.9067	**0.9186**
	10	0.9238	0.9397	0.9263	0.9591	0.9537	0.9549	0.9373	0.9542	0.9590	0.9589	0.9278	**0.9592**
	14	0.9443	0.9564	0.9459	0.9543	0.9522	0.9587	0.9433	0.9565	0.9623	0.9645	0.9367	**0.9654**
	18	0.9598	0.9662	0.9627	0.9760	0.9749	0.9796	0.9627	0.9763	0.9791	0.9814	0.9523	**0.9830**
	22	0.9712	0.9763	0.9677	0.9801	0.9799	0.9801	0.9787	0.9823	0.9810	**0.9859**	0.9789	0.9855
	26	0.9751	0.9801	0.9689	0.9853	0.9830	0.9873	0.9769	0.9853	0.9833	0.9894	0.9815	**0.9900**
Image2	6	0.8472	0.8606	0.8567	0.8648	0.8870	0.8812	0.8571	0.8634	0.8781	**0.8896**	0.8688	0.8868
	10	0.9148	0.9463	0.9311	0.9461	0.9516	0.9608	0.9338	0.9473	0.9586	0.9442	0.9298	**0.9622**
	14	0.9382	0.9633	0.9672	0.9716	0.9780	0.9779	0.9661	0.9745	0.9737	0.9765	0.9560	**0.9808**
	18	0.9531	0.9621	0.9688	0.9798	0.9715	0.9769	0.9703	0.9789	0.9766	0.9769	0.9623	**0.9882**
	22	0.9665	0.9714	0.9728	0.9821	0.9858	0.9899	0.9743	0.9865	0.9855	0.9802	0.9676	**0.9908**
	26	0.9736	0.9819	0.9811	0.9898	0.9805	0.9921	0.9614	0.9912	0.9910	0.9898	0.9663	**0.9924**
Image3	6	0.9011	0.9003	0.8939	0.9054	0.9078	0.9059	0.8748	0.8001	0.9061	0.9074	0.8998	**0.9165**
	10	0.9351	0.9377	0.9378	0.9537	0.9473	0.9579	0.9234	0.8724	0.9590	**0.9677**	0.9358	**0.9677**
	14	0.9651	0.9658	0.9657	**0.9827**	0.9784	0.9820	0.9614	0.9053	0.9621	0.9816	0.9564	0.9812
	18	0.9688	0.9763	0.9783	0.9890	0.9849	0.9884	0.9684	0.9198	0.9668	0.9850	0.9647	**0.9892**
	22	0.9780	0.9817	0.9826	0.9912	0.9841	0.9911	0.9715	0.9194	0.9720	**0.9933**	0.9723	0.9928
	26	0.9805	0.9873	0.9897	0.9920	0.9886	0.9933	0.9770	0.9208	0.9854	0.9951	0.9790	**0.9955**
Image4	6	0.9088	0.9141	0.9140	0.9128	0.9175	0.9135	0.9110	0.9175	0.9116	0.9106	0.9102	**0.9198**
	10	0.9378	0.9437	0.9401	0.9545	0.9522	0.9512	0.9403	0.9457	0.9418	0.9532	0.9311	**0.9558**
	14	0.9522	0.9544	0.9573	0.9779	0.9721	0.9699	0.9687	0.9839	0.9830	0.9831	0.9662	**0.9849**
	18	0.9737	0.9762	0.9758	0.9911	0.9844	0.9867	0.9800	0.9901	0.9874	0.9886	0.9699	**0.9918**
	22	0.9872	0.9862	0.9819	0.9941	0.9901	0.9941	0.9787	0.9923	0.9911	0.9940	0.9762	**0.9944**
	26	0.9857	0.9880	0.9791	0.9955	0.9917	0.9953	0.9872	0.9956	0.9946	0.9946	0.9787	**0.9956**
Image5	6	0.9082	0.9082	0.8523	0.9072	0.9055	0.9070	0.9078	0.8867	0.7758	0.9079	0.9055	**0.9219**
	10	0.8911	0.9101	0.8831	0.9519	0.9552	0.9485	0.9388	0.8895	0.8476	0.9432	0.9134	**0.9601**
	14	0.9575	0.9576	0.9678	**0.9800**	0.9700	0.9623	0.9412	0.8933	0.8528	0.9770	0.9394	0.9744
	18	0.9705	0.9612	0.9727	0.9820	0.9734	0.9697	0.9712	0.9411	0.8832	0.9780	0.9532	**0.9824**
	22	0.9720	0.9745	0.9798	**0.9880**	0.9783	0.9717	0.9732	0.9424	0.9000	0.9810	0.9680	0.9856
	26	0.9731	0.9814	0.9825	**0.9900**	0.9832	0.9771	0.9783	0.9477	0.9018	0.9895	0.9712	**0.9900**
Image6	6	0.8191	0.8124	0.8138	0.8102	0.8147	0.8176	0.7919	0.7783	0.7527	0.8201	0.7034	**0.8240**
	10	0.8034	0.8697	0.8491	0.8501	0.8552	0.8843	0.8158	0.7800	0.8078	0.8461	0.7265	**0.8951**
	14	0.8177	0.8837	0.8804	0.8907	0.8751	0.9053	0.8371	0.7826	0.8290	0.8887	0.8236	**0.9086**
	18	0.8855	0.8993	0.8810	0.9027	0.8762	0.9101	0.8509	0.8078	0.9309	0.9054	0.8433	**0.9189**
	22	0.9246	0.9289	0.8829	0.9318	0.8992	0.9248	0.8834	0.8356	0.8401	0.9290	0.8836	**0.9322**
	26	0.9559	0.9593	0.8885	0.9570	0.9278	0.9345	0.9503	0.9397	0.9346	0.9560	0.9014	**0.9574**
Average		0.9335	0.9435	0.9354	0.9524	0.9479	0.9526	0.9345	0.9192	0.9267	0.9534	0.9252	**0.9594**

**Table 9:** The NCC results of standard image segmentation using hybrid fitness function for all algorithms

Image	*K*	Algorithm											
		RSA	SOA	BWOA	MPA	AO	SMA	AOA	JOA	MFO	HHO	SCA	Proposed COVID
Image1	6	0.9777	0.9782	0.9820	0.9890	0.9938	0.9892	0.9883	0.9895	**0.9905**	0.9879	0.9835	**0.9905**
	10	0.9889	0.9926	0.9920	**0.9960**	0.9955	**0.9960**	0.9944	0.9956	0.9960	0.9959	0.9940	**0.9960**
	14	0.9920	0.9944	0.9935	0.9974	0.9968	0.9965	0.9959	0.9967	0.9973	0.9978	0.9945	**0.9980**
	18	0.9960	0.9972	0.9962	0.9980	0.9980	0.9982	0.9964	0.9979	0.9982	0.9983	0.9958	**0.9985**
	22	0.9966	0.9979	0.9968	0.9983	0.9982	0.9985	0.9975	0.9980	0.9965	0.9986	0.9969	**0.9989**
	26	0.9975	0.9984	0.9972	0.9987	0.9985	0.9990	0.9981	0.9984	0.9988	0.9990	0.9982	**0.9991**
Image2	6	0.9844	**0.9878**	0.9872	0.9875	0.9874	0.9876	0.9831	0.9866	0.9868	0.9860	0.9859	0.9872
	10	0.9920	0.9936	0.9930	0.9936	0.9936	0.9932	0.9915	0.9939	0.9937	0.9937	0.9933	**0.9943**
	14	0.9936	0.9961	0.9954	0.9973	0.9967	0.9975	0.9955	0.9975	**0.9976**	**0.9976**	0.9951	**0.9976**
	18	0.9952	0.9968	0.9960	0.9977	0.9972	0.9982	0.9960	0.9980	0.9981	0.9981	0.9964	**0.9984**
	22	0.9970	0.9975	0.9967	0.9981	0.9980	**0.9989**	0.9969	0.9984	0.9987	0.9979	0.9955	**0.9989**
	26	0.9956	0.9981	0.9978	0.9986	0.9979	**0.9992**	0.9964	0.9989	0.9987	0.9990	0.9970	**0.9992**
Image3	6	0.9707	0.9707	0.9655	0.9731	0.9734	0.9732	0.9626	0.9320	0.9728	0.9728	0.9686	**0.9769**
	10	**0.9870**	0.9802	0.9771	0.9842	0.9828	0.9842	0.0801	0.9539	0.9840	0.9835	0.9799	0.9843
	14	0.9909	0.9903	0.9912	**0.9946**	0.9932	0.9946	0.9884	0.9735	0.9875	0.9943	0.9892	0.9945
	18	0.9912	0.9930	0.9936	0.9965	0.9953	0.9963	0.9907	0.9745	0.9890	0.9953	0.9910	**0.9967**
	22	0.9932	0.9953	0.9960	0.9968	0.9961	0.9969	0.9932	0.9756	0.9933	0.9968	0.9926	**0.9970**
	26	0.9935	0.9961	0.9967	0.9973	0.9964	0.9977	0.9945	0.9789	0.9966	0.9980	0.9939	**0.9979**
Image4	6	0.9740	0.9733	0.9722	0.9717	0.9735	0.9725	0.9718	0.9734	0.9718	0.9714	0.9711	**0.9742**
	10	0.9815	0.9862	0.9871	0.9834	0.9855	0.9867	0.9823	0.9846	0.9860	0.9880	0.9823	**0.9884**
	14	0.9901	0.9921	0.9924	0.9948	0.9944	**0.9959**	0.9930	0.9954	0.9954	0.9954	0.9908	0.9957
	18	0.9927	0.9944	0.9943	0.9966	0.9952	0.**9970**	0.9936	0.9966	0.9964	0.9967	0.9936	**0.9970**
	22	0.9956	0.9957	0.9950	0.9979	0.9968	**0.9981**	0.9938	0.9976	0.9977	0.9980	0.9948	0.9980
	26	0.9955	0.9966	0.9940	0.9984	0.9974	**0.9985**	0.9967	0.9982	0.9983	0.9984	0.9954	**0.9985**
Image5	6	0.9847	0.9845	0.9663	0.9828	0.9819	0.9845	0.9834	0.9697	0.9002	0.9833	0.9754	**0.9862**
	10	0.9889	0.9902	0.9912	**0.9924**	0.9912	0.9910	0.9883	0.9747	0.9281	0.9897	0.9815	**0.9924**
	14	0.9921	0.9938	0.9940	**0.9963**	0.9951	0.9924	0.9914	0.9795	0.9577	0.9957	0.9753	**0.9963**
	18	0.9936	0.9943	0.9944	0.9966	0.9950	0.9930	0.9934	0.9824	0.9678	0.9960	0.9798	**0.9968**
	22	0.9943	0.9953	0.9956	0.9970	0.9961	0.9945	0.9953	0.9874	0.9754	0.9970	0.9862	**0.9972**
	26	0.9957	0.9965	0.9967	0.9980	0.9972	0.9957	0.9964	0.9910	0.9801	**0.9979**	0.9938	0.9976
Image6	6	0.9732	0.9782	0.9758	0.9761	0.9765	0.9701	0.9710	0.9705	0.9673	0.9754	0.8635	**0.9788**
	10	0.957	0.9778	0.9769	0.9730	0.9775	0.9748	0.9731	0.9722	0.9712	0.9767	0.8718	**0.9819**
	14	0.9785	0.9794	0.9791	0.9778	0.9789	0.9787	0.9755	0.9734	0.9779	0.9786	0.9807	**0.9870**
	18	0.9860	0.9884	0.9842	0.9797	0.9760	0.9862	0.9826	0.9847	0.9856	0.9876	0.9852	**0.9924**
	22	0.9950	0.9951	0.9867	0.9945	0.9912	0.9938	0.9944	0.9933	0.9932	0.9950	0.9883	**0.9953**
	26	0.9977	**0.9976**	0.9885	0.9974	0.9937	0.9958	0.9974	0.9958	0.9959	0.9972	0.9936	0.9974
Average		0.9888	0.9904	0.9891	0.9915	0.9911	0.9914	0.9642	0.9849	0.9838	0.9918	0.9817	**0.9932**

**Table 10:** The fitness results of satellite image segmentation using hybrid fitness function for all algorithms

Image	*K*	Algorithm											
		RSA	SOA	BWOA	MPA	AO	SMA	AOA	JOA	MFO	HHO	SCA	Proposed COVID
Sat_img1	6	873.5	881.3	857.0	883.1	882.1	**883.3**	879.7	883.1	883.2	883.1	874.1	**883.3**
	10	899.3	915.3	917.3	921.0	919.3	921.6	899.3	921.5	921.7	920.5	910.1	**921.9**
	14	916.8	925.6	930.7	933.8	934.1	935.8	932.1	935.8	**936.6**	936.4	924.5	936.5
	18	935.2	939.2	936.9	**945.6**	944.7	945.1	934.3	944.7	945.2	944.7	935.7	945.5
	22	940.3	944.5	942.4	949.5	948.3	950.4	939.4	950.3	951.4	952.1	942.4	**951.9**
	26	946.2	950.0	949.7	954.0	954.1	955.1	947.7	955.4	956.5	955.9	946.9	**956.9**
Sat_img2	6	866.5	874.1	876.2	**876.5**	875.1	872.5	872.6	874.5	875.2	875.3	871.1	876.1
	10	876.3	890.4	892.5	897.1	893.8	891.5	885.5	892.5	**893.6**	894.2	886.3	895.5
	14	898.1	901.9	904.1	**905.9**	905.3	901.5	897.2	902.5	905.5	905.8	898.2	**905.9**
	18	906.7	909.9	909.2	911.2	911.4	906.3	904.0	905.5	909.2	908.6	904.9	**911.8**
	22	912.4	914.3	916.4	917.6	918.3	916.3	911.4	910.4	915.6	**918.5**	915.1	**918.5**
	26	916.4	918.1	920.1	924.6	921.7	920.5	916.7	916.5	920.6	923.8	917.2	**922.5**
Sat_img3	6	1878.5	1900.2	1891.5	1904.1	**1903.8**	1903.5	1900.9	1903.7	**1903.8**	**1903.8**	1895.1	**1903.8**
	10	1923.4	1922.1	1933.2	1933.5	1933.3	1931.6	1924.4	1931.5	1932.5	1931.6	1923.5	**1933.8**
	14	**1958.4**	1951.3	1948.3	1957.2	1951.3	1957.1	1944.3	1952.8	1957.1	1955.9	1940.9	1957.5
	18	1957.8	1961.4	1959.6	1965.9	1963.4	**1966.5**	1952.4	1964.7	1966.2	1965.7	1960.5	**1966.5**
	22	1962.5	1967.4	1968.3	1972.6	1972.5	1972.8	1960.5	1972.1	1972.5	1972.6	1.9652	**1973.5**
	26	1969.7	1971.8	1972.3	1975.6	1976.9	1977.7	1968.7	1977.3	1977.6	1977.2	1971.1	**1978.1**
Sat_img4	6	4329.4	4328.9	4329.4	4332.4	4330.1	4332.3	4321.9	4332.3	4332.4	4332.2	4326.1	**4332.7**
	10	4357.4	4355.4	4358.4	4363.3	4361.4	4366.2	4352.6	4366.3	4366.4	4368.0	4359.3	**4366.7**
	14	4373.5	4375.6	4375.7	4380.1	4377.4	4380.9	4368.7	4380.1	4381.3	4380.2	4372.5	**4381.7**
	18	4380.6	4384.7	4378.6	4387.8	4386.2	4387.1	4380.9	4388.5	4388.5	**4389.7**	4382.8	4388.7
	22	4388.4	4390.4	4389.1	4393.6	4392.5	4394.4	4388.5	4394.5	4394.7	4394.7	4387.8	**4395.0**
	26	4391.2	4396.6	4390.8	4397.6	4397.3	4399.4	4390.7	4399.2	4399.5	4399.5	4392.8	**4399.9**
Sat_img5	6	1061.2	1075.9	1074.5	1077.4	1077.3	1077.4	1073.3	1077.2	1077.4	**1077.5**	1069.9	1077.4
	10	1104.4	1108.3	1111.3	1115.8	1115.5	1115.5	1108.4	1115.5	1115.7	1115.8	1104.0	**1116.1**
	14	1118.3	1127.4	1123.8	1129.4	1130.3	1131.0	1119.6	1130.1	1130.5	1131.1	1123.0	**1131.5**
	18	1127.9	1135.4	1133.9	1138.5	1137.6	1148.4	1131.0	**1139.5**	1139.4	1139.1	1134.1	**1139.5**
	22	1124.4	1141.6	1142.4	1145.1	1144.3	**1146.3**	1141.4	1145.3	1145.4	1146.1	1136.9	1145.9
	26	1136.3	1144.5	1146.4	1147.9	1147.6	1150.4	1142.7	1150.4	1150.4	1150.6	1140.8	**1150.9**
Sat_img6	6	1672.4	1682.3	1672.5	1682.8	1682.4	1682.7	1677.9	1682.6	**1682.8**	1682.3	1643.4	**1682.8**
	10	1722.4	1727.3	1724.3	1730.6	1730.1	1731.1	1728.4	1731.1	1731.2	1730.5	1712.3	**1733.3**
	14	1731.4	1739.4	1736.0	1744.0	1745.9	1747.2	1737.0	1746.5	1747.1	1747.1	1740.1	**1747.5**
	18	1742.3	1750.5	1752.2	1755.8	1752.4	1754.3	1751.7	1755.9	1756.1	1755.3	1743.3	**1757.4**
	22	1748.4	1758.4	1760.3	1763.3	1762.8	1763.3	1762.5	1765.1	1762.4	1762.6	1751.7	**1767.3**
	26	1758.0	1761.6	1760.3	1765.9	1764.2	1764.5	1756.6	1767.0	1768.3	**1768.8**	1759.1	**1768.8**
Average		1827.9	1833.9	1832.9	1838.3	1837.3	1838.3	1830.6	1837.8	1838.7	1838.7	1774.9	**1839.5**

**Table 11:** The MSE results of satellite image segmentation using hybrid fitness function for all algorithms

Image	*K*	Algorithm											
		RSA	SOA	BWOA	MPA	AO	SMA	AOA	JOA	MFO	HHO	SCA	Proposed COVID
Sat_img1	6	181.84	164.55	177.03	164.67	158.65	164.18	164.10	166.31	164.29	163.37	171.23	**162.03**
	10	139.45	132.66	130.64	131.75	127.45	122.86	142.75	120.12	123.54	125.3	142.45	**120.43**
	14	107.57	78.34	80.80	83.06	86.68	70.67	82.12	**68.74**	72.75	71.69	94.54	69.85
	18	92.43	48.83	77.56	49.60	52.40	46.54	85.02	52.49	47.06	49.34	88.75	**41.31**
	22	78.45	37.35	65.34	38.34	44.34	36.45	66.75	39.33	38.56	35.34	71.45	**33.55**
	26	59.46	39.20	51.07	32.64	35.65	24.91	45.86	30.87	24.43	27.20	45.59	**23.53**
Sat_img2	6	144.07	146.96	139.53	146.90	155.61	157.35	**142.55**	223.21	155.38	156.45	159.87	150.54
	10	124.65	98.45	133.65	102.35	99.34	95.34	124.35	198.35	98.34	112.20	120.35	**90.73**
	14	95.16	68.54	104.21	59.34	75.69	63.54	95.26	168.97	61.45	71.31	74.82	**56.05**
	18	73.40	45.52	53.85	**39.72**	61.91	52.45	83.96	142.86	48.54	64.47	116.32	44.45
	22	63.86	47.46	46.34	**32.64**	55.03	35.74	66.34	114.45	32.95	42.64	65.35	32.72
	26	36.29	30.32	38.71	27.21	30.38	24.35	53.43	82.82	22.54	26.43	33.16	**20.90**
Sat_img3	6	188.55	190.39	194.96	191.66	190.21	188.93	194.34	189.83	191.69	189.09	198.54	**185.59**
	10	142.75	148.34	142.39	135.92	140.46	139.53	146.75	171.47	140.43	142.53	153.72	**129.79**
	14	117.85	103.66	127.85	86.45	85.75	85.34	121.5	110.75	80.82	96.41	87.45	**79.11**
	18	86.18	75.53	81.69	59.89	66.59	66.34	95.75	69.66	**53.14**	62.76	86.49	58.15
	22	53.75	59.45	64.84	46.45	46.74	39.65	87.84	47.45	39.28	38.65	69.56	**35.63**
	26	51.95	48.07	50.70	36.69	36.20	32.64	74.44	37.65	31.46	30.96	53.71	**27.93**
Sat_img4	6	113.67	109.36	107.26	113.24	109.24	113.62	105.58	112.69	113.13	113.16	111.65	**102.29**
	10	93.54	82.64	85.67	95.35	78.45	82.64	87.46	77.854	78.56	80.65	83.99	**75.76**
	14	76.40	60.74	60.95	77.98	55.83	60.15	62.675	59.067	56.17	59.66	66.83	**53.71**
	18	55.92	53.66	57.45	41.57	37.93	39.56	58.004	38.46	35.65	39.67	46.09	**35.27**
	22	37.57	32.54	48.71	36.34	32.65	**22.90**	44.76	26.99	25.82	28.13	41.64	23.58
	26	37.44	29.09	38.32	23.44	27.56	22.67	35.54	21.06	18.09	20.63	34.10	**17.68**
Sat_img5	6	**147.57**	164.31	163.32	163.87	164.81	163.50	162.83	163.44	163.87	163.93	165.98	160.67
	10	131.75	127.46	131.76	128.57	111.75	125.75	133.65	123.65	114.64	112.05	122.64	**110.76**
	14	87.09	71.28	85.28	77.56	**65.78**	69.42	95.72	72.87	67.19	72.56	80.36	66.84
	18	75.28	61.25	68.64	49.11	56.61	44.28	81.18	48.19	48.75	51.84	60.78	**43.27**
	22	64.76	52.64	47.45	44.75	42.56	**31.26**	65.74	41.45	38.65	38.35	55.34	35.75
	26	52.40	39.74	38.61	40.88	31.95	25.74	52.71	26.71	25.80	26.36	46.90	**22.86**
Sat_img6	6	172.75	177.65	179.01	177.92	177.62	177.01	180.65	178.00	177.95	175.99	174.21	**172.04**
	10	154.64	122.64	143.65	136.74	120.6	134.6	127.45	119.56	143.54	112.68	140.68	**104.54**
	14	109.67	96.07	104.66	98.21	85.33	82.48	110.59	84.18	88.45	85.34	128.56	**80.47**
	18	79.60	76.08	62.07	64.73	57.85	59.67	73.04	60.07	55.61	60.66	99.77	**51.60**
	22	57.46	56.75	57.45	47.86	41.44	40.79	70.345	45.76	33.65	35.65	69.34	**32.72**
	26	48.38	49.35	50.64	38.38	31.45	33.54	67.82	31.01	29.34	28.55	58.21	**28.41**
Average		95.37	84.07	91.44	81.16	79.95	77.17	96.91	93.50	76.15	78.11	95.01	**71.68**

**Table 12:** The PSNR results of satellite image segmentation using hybrid fitness function for all algorithms

Image	k	Algorithm											
		RSA	SOA	BWOA	MPA	AO	SMA	AOA	JOA	MFO	HHO	SCA	Proposed COVID
Sat_img1	6	21.49	22.57	19.43	22.33	22.14	22.42	21.17	22.60	22.33	**22.88**	21.76	**22.88**
	10	22.76	23.65	23.77	24.21	23.86	25.14	23.65	25.48	25.53	25.44	24.34	**25.65**
	14	25.33	27.34	28.22	28.58	28.51	29.43	27.94	29.42	29.47	29.33	26.96	**29.58**
	18	26.46	30.86	28.90	31.17	30.82	31.38	28.26	30.88	31.34	31.14	28.16	**31.87**
	22	28.54	30.54	29.544	30.17	30.67	32.10	29.89	31.76	32.39	32.48	28.75	**32.54**
	26	30.11	32.18	30.97	32.97	32.59	34.09	31.28	33.23	34.25	33.78	30.79	**35.16**
Sat_img2	6	21.351	20.52	20.36	20.58	**20.79**	20.37	20.19	16.35	19.76	20.58	20.17	20.64
	10	22.54	25.35	23.64	27.22	25.35	26.98	21.53	16.76	26.76	25.96	25.34	**27.49**
	14	24.701	29.40	25.96	30.45	28.97	30.46	22.90	17.62	29.33	28.59	27.41	**30.50**
	18	29.16	31.34	30.48	32.81	27.67	32.23	27.60	23.62	32.54	30.69	24.16	**32.75**
	22	30.53	32.14	29.54	32.95	31.45	32.55	28.94	25.76	32.67	32.24	30.54	**32.96**
	26	32.20	33.21	31.74	**35.77**	33.29	34.65	30.57	27.41	34.34	32.90	32.74	34.45
Sat_img3	6	18.39	19.51	18.59	19.06	19.25	19.33	18.67	19.25	19.15	19.29	19.04	**19.59**
	10	21.56	23.87	**24.14**	23.24	21.64	23.88	22.64	20.81	23.81	23.56	22.99	24.04
	14	25.75	27.45	26.74	28.43	28.21	27.53	25.49	27.07	27.39	28.02	25.74	**28.58**
	18	27.89	29.11	28.56	30.29	29.53	30.31	26.86	29.30	30.25	30.05	28.42	**30.33**
	22	28.54	30.54	29.45	30.85	31.27	32.28	28.16	31.67	32.10	31.75	29.35	**32.77**
	26	30.80	31.20	30.94	32.48	32.54	33.14	28.91	32.56	32.56	33.19	30.56	**33.66**
Sat_img4	6	23.19	22.69	23.58	23.01	23.06	22.98	22.16	23.07	23.06	23.03	23.54	**24.13**
	10	25.92	25.45	25.58	26.46	26.72	27.14	25.84	26.83	27.22	25.35	26.31	**27.45**
	14	27.55	28.75	28.88	29.76	29.84	30.03	27.86	29.76	30.26	29.80	27.38	**30.31**
	18	29.30	30.04	29.60	31.43	31.44	32.27	29.64	**32.62**	32.54	32.34	29.96	32.61
	22	29.93	31.34	30.35	32.49	31.45	33.16	30.39	33.77	33.80	33.45	29.46	**33.86**
	26	31.81	33.35	31.07	34.32	33.62	34.56	32.23	34.89	**35.35**	34.94	32.37	35.19
Sat_img5	6	22.42	22.65	22.65	22.59	22.58	22.64	22.49	22.72	22.59	22.59	21.39	**22.94**
	10	26.12	26.85	26.22	26.24	25.47	25.35	25.71	26.35	26.87	26.87	25.38	**26.99**
	14	26.79	29.23	28.15	29.01	29.74	29.53	27.17	27.22	**29.57**	28.01	27.88	28.88
	18	28.59	29.94	29.39	31.20	30.52	31.60	28.26	28.29	31.22	30.92	29.90	**31.63**
	22	28.15	30.14	31.87	31.61	32.10	33.18	28.47	29.34	32.24	31.34	28.45	**32.56**
	26	30.53	32.03	32.25	32.01	33.08	33.60	30.68	31.22	34.01	33.92	30.73	**34.71**
Sat_img6	6	21.29	21.44	21.22	21.43	21.49	21.51	21.09	21.47	21.43	21.69	21.27	**21.97**
	10	22.56	22.48	22.36	23.21	23.24	23.44	22.65	22.65	24.42	24.45	23.76	**25.45**
	14	25.65	27.34	26.40	28.02	28.46	28.54	26.61	28.66	29.11	28.87	25.33	**29.36**
	18	28.00	28.71	30.04	30.01	29.67	30.76	28.25	30.32	30.70	30.24	26.96	**30.97**
	22	30.68	31.52	30.82	31.42	31.94	32.35	29.64	32.25	32.78	32.34	27.89	**32.95**
	26	31.19	30.93	30.70	32.28	32.65	33.26	30.09	33.20	33.52	33.22	29.89	**33.93**
Average		26.60	27.93	27.28	28.61	28.21	29.00	26.49	27.11	29.07	28.75	26.80	**29.48**

**Table 13:** The SSIM results of satellite image segmentation using hybrid fitness function for all algorithms

Image	*K*	Algorithm											
		RSA	SOA	BWOA	MPA	AO	SMA	AOA	JOA	MFO	HHO	SCA	Proposed COVID
Sat_img1	6	0.9052	0.9205	0.8417	0.9151	0.9041	0.9173	0.8817	0.9201	0.9152	0.9189	0.9031	**0.9223**
	10	0.9247	0.9533	0.9178	0.9511	0.9533	0.9535	0.9433	0.9533	0.9532	0.9524	0.9356	**0.9545**
	14	0.9423	0.9712	0.9688	0.9733	0.9724	**0.9770**	0.9662	0.9761	0.9765	0.9769	0.9620	**0.9770**
	18	0.9533	0.9810	0.9700	0.9827	0.9810	0.9860	0.9696	0.9820	0.9842	0.9830	0.9638	**0.9862**
	22	0.9714	0.9822	0.9734	0.9844	0.9829	**0.9889**	0.9786	0.9843	0.9884	0.9858	0.9784	**0.9889**
	26	0.9750	0.9853	0.9795	0.9890	0.9858	0.9910	0.9825	0.9866	0.9914	0.9898	0.9839	**0.9915**
Sat_img2	6	0.7648	0.7692	0.7514	0.7685	0.7567	0.7623	0.7365	0.5053	0.7487	0.7532	**0.7951**	0.7852
	10	0.8254	0.9154	0.8429	0.9233	0.8534	0.9233	0.9143	0.5567	0.9229	0.9000	0.8745	**0.9239**
	14	0.8660	0.9396	0.8934	**0.9523**	0.9358	0.9520	0.8362	0.5915	0.9513	0.9382	0.9041	**0.9523**
	18	0.9344	0.9604	0.9514	0.9612	0.9460	0.9600	0.9153	0.8328	0.9610	0.9423	0.9201	**0.9614**
	22	0.9536	0.9645	0.9533	**0.9687**	0.9526	0.9621	0.9433	0.8954	0.9681	0.9568	0.9436	**0.9687**
	26	0.9640	0.9688	0.9585	**0.9817**	0.9698	0.9755	0.9578	0.9210	0.9772	0.9737	0.9695	0.9802
Sat_img3	6	0.7435	0.7849	0.7591	0.7726	0.7772	0.7783	0.7607	0.7770	0.7751	0.7780	0.7665	**0.7859**
	10	0.8472	0.8573	0.8282	**0.8654**	0.8282	0.8544	0.8435	0.8562	0.8512	0.8521	0.9033	0.8632
	14	0.9423	0.9567	0.9412	0.9627	0.9563	0.9612	0.9412	0.9547	0.9388	**0.9641**	0.9243	0.9636
	18	0.9563	0.9689	0.9593	0.9730	0.9700	0.9688	0.9476	0.9589	0.9733	0.9689	0.9661	**0.9741**
	22	0.9599	0.9722	0.9731	0.9779	0.9802	0.9745	0.9525	0.9687	0.9826	0.9765	0.9688	**0.9833**
	26	0.9646	0.9784	0.9755	0.9837	0.9832	0.9840	0.9557	0.9783	0.9840	0.9833	0.9712	**0.9859**
Sat_img4	6	0.7281	0.7566	0.7544	0.7518	0.7554	0.7515	0.7335	0.7525	0.7517	0.7519	0.7645	**0.7735**
	10	0.7548	0.8077	0.8167	0.8222	0.8132	0.7956	0.8067	0.8244	0.8154	0.8189	0.8224	**0.8245**
	14	0.7963	0.8418	**0.8787**	0.8563	0.8537	0.8614	0.8547	0.8611	0.8544	0.8582	0.8577	0.8687
	18	0.8330	0.8872	0.8823	0.9346	0.9307	0.9328	0.9271	0.9322	0.8867	0.9245	0.8635	**0.9371**
	22	0.8842	0.9247	0.8875	0.9464	0.9453	0.9455	0.9294	**0.9491**	0.9041	0.9315	0.8756	0.9467
	26	0.9146	0.9387	0.9113	**0.9540**	0.9501	0.9568	0.9324	0.9605	0.9510	0.9512	0.8861	0.9523
Sat_img5	6	0.9198	0.9164	0.9137	0.9136	0.9150	0.9156	0.9103	0.9168	0.9146	0.9147	0.8925	**0.9205**
	10	0.9354	0.9588	0.9487	0.9604	0.9611	0.9615	0.9522	0.9606	0.9612	0.9613	0.9447	**0.9617**
	14	0.9587	0.9730	0.9684	0.9807	0.9776	0.9810	0.9645	0.9778	**0.9815**	0.9778	0.9689	0.9787
	18	0.9722	0.9813	0.9791	0.9870	0.9846	**0.9870**	0.9747	0.9851	0.9854	0.9853	0.9806	**0.9867**
	22	0.9752	0.9852	0.9821	**0.9898**	0.9857	0.9895	0.9894	0.9862	0.9885	0.9889	0.9855	**0.9898**
	26	0.9767	0.9894	0.9866	0.9903	0.9896	0.9905	0.9820	0.9870	0.9903	**0.9928**	0.9836	0.9925
Sat_img6	6	0.8250	0.8360	0.8248	0.8333	0.8359	0.8363	0.8340	0.8348	0.8331	0.8358	0.8377	**0.8445**
	10	0.8993	0.8845	0.8867	0.9101	0.8845	0.9101	0.9110	0.8973	0.9021	0.9100	0.9057	**0.9115**
	14	0.9446	0.9149	0.9297	0.9448	0.9403	0.9380	0.9314	0.9361	0.9368	0.9405	0.9342	**0.9489**
	18	0.9620	0.9417	0.9612	0.9515	0.9476	0.9646	0.9348	0.9610	0.9730	0.9732	0.9551	**0.9759**
	22	0.9664	0.9642	0.9642	0.9812	0.9577	0.9810	0.9380	0.9802	0.9798	0.9811	0.9655	**0.9825**
	26	0.9682	0.9620	0.9673	0.9840	0.9586	0.9822	0.9447	0.9817	0.9828	0.9829	0.9724	**0.9845**
Average		0.9057	0.9248	0.9133	0.9327	0.9243	0.9319	0.9132	0.8967	0.9287	0.9298	0.9175	**0.9369**

**Table 14:** The FSIM results of satellite image segmentation using hybrid fitness function for all algorithms

Image	*K*	Algorithm											
		RSA	SOA	BWOA	MPA	AO	SMA	AOA	JOA	MFO	HHO	SCA	Proposed COVID
Sat_img1	6	0.9548	0.9517	0.9109	0.9476	0.9428	0.9499	0.9251	0.9584	0.9459	0.9579	0.9500	**0.9601**
	10	0.9662	0.9657	0.9645	0.9677	0.9684	0.9678	0.9467	0.9687	0.9756	0.9760	0.9674	**0.9768**
	14	0.9719	0.9823	0.9730	0.9861	0.9887	0.9882	0.9816	0.9882	0.9887	0.9883	0.9738	**0.9892**
	18	0.9781	0.9898	0.9888	0.9929	0.9910	0.9928	0.9839	0.9919	0.9916	0.9931	0.9831	**0.9935**
	22	0.9865	0.9917	0.9895	0.9934	0.9932	0.9943	0.9857	0.9926	0.9933	0.9952	0.9854	**0.9954**
	26	0.9900	0.9934	0.9916	0.9944	0.9948	0.9960	0.9889	0.9952	**0.9964**	0.9962	0.9878	**0.9964**
Sat_img2	6	0.8514	0.8934	0.8862	0.8921	0.8807	**0.8939**	0.8775	0.6590	0.8922	0.8865	0.8800	0.8934
	10	0.9070	0.9378	0.9178	0.9418	0.9264	**0.9422**	0.8878	0.6879	0.9415	0.9320	0.8875	0.9420
	14	0.9227	0.9595	0.9339	0.9579	0.9576	0.9656	0.8930	0.7015	0.9641	0.9496	0.9067	**0.9659**
	18	0.9523	0.9720	0.9633	0.9714	0.9451	0.9733	0.9407	0.8977	0.9711	0.9625	0.9136	**0.9737**
	22	0.9657	0.9744	0.9694	0.9724	0.9577	0.9792	0.9487	0.9154	0.9726	0.9702	0.9564	**0.9795**
	26	0.9699	0.9791	0.9747	**0.9858**	0.9728	0.9824	0.9587	0.9230	0.9788	0.9768	0.9799	0.9844
Sat_img3	6	0.8922	0.9095	0.9011	0.9025	0.9113	0.9180	0.8967	0.9136	0.9028	0.9149	0.8982	**0.9193**
	10	0.9320	0.9339	**0.9581**	0.9369	0.9340	0.9368	0.9243	0.9282	0.9322	0.9345	0.9495	0.9499
	14	0.9553	0.9722	0.9747	0.9736	0.9744	0.9722	0.9658	0.9759	0.9650	0.9723	0.9664	**0.9764**
	18	0.9725	0.9834	0.9799	0.9863	0.9857	0.9867	0.9687	0.9826	0.9841	0.9860	0.9814	**0.9879**
	22	0.9767	0.9867	0.9867	0.9906	0.9912	0.9910	0.9786	0.9879	0.9910	0.9902	0.843	**0.9915**
	26	0.9799	0.9883	0.9891	0.9921	**0.9925**	0.9919	0.9826	0.9899	0.9921	0.9922	0.9867	**0.9925**
Sat_img4	6	0.9004	0.9036	0.9024	0.8939	0.9040	0.8931	0.8921	0.8985	0.8934	0.8939	0.9043	**0.9193**
	10	0.9187	0.9243	0.9136	0.9231	0.9243	0.9490	0.9265	0.9511	0.9500	0.9512	0.9449	**0.9520**
	14	0.9243	0.9345	0.9368	0.9453	0.9534	0.9732	0.9522	0.9750	0.9760	**0.9762**	0.9654	0.9758
	18	0.9642	0.9731	0.9698	0.9834	0.9818	0.9844	0.9645	0.9853	**0.9855**	0.9846	0.9713	**0.9855**
	22	0.9668	0.9822	0.9712	0.9845	0.9858	0.9890	0.9743	0.9884	0.9884	0.9874	0.9786	**0.9895**
	26	0.9752	0.9868	0.9761	0.9894	0.9872	0.9867	0.9820	**0.9905**	0.9902	0.9902	0.9819	**0.9905**
Sat_img5	6	0.9194	0.9377	0.9373	0.9332	0.9375	0.9342	0.9269	0.9402	0.9332	0.9334	0.9239	**0.9424**
	10	0.9356	0.9668	0.9654	0.9636	0.9668	0.9675	0.9543	0.9678	0.9688	0.9703	0.9432	**0.9710**
	14	0.9585	0.9763	0.9700	0.9849	0.9810	0.9848	0.9691	0.9818	0.9854	0.9855	0.9678	**0.9857**
	18	0.9725	0.9830	0.9828	**0.9898**	0.9890	0.9895	0.9830	0.9864	0.9895	0.9895	0.9837	**0.9898**
	22	0.9832	0.9886	0.9857	0.9912	0.9899	0.9905	0.9844	0.9882	0.9900	0.9912	0.9846	**0.9914**
	26	0.9889	0.9917	0.9879	0.9942	0.9905	0.9936	0.9857	0.9889	0.9905	**0.9942**	0.9854	0.9941
Sat_img6	6	0.9169	0.9194	0.9110	0.9145	0.9198	0.9166	0.9140	0.9183	0.9140	0.9232	0.9176	**0.9267**
	10	0.9345	0.9215	0.9227	0.9437	0.9336	0.9389	0.9367	0.9375	0.9391	0.9455	0.9457	**0.9480**
	14	0.9677	0.9611	0.9662	0.9723	0.9733	**0.9753**	0.9703	0.9727	0.9710	0.9729	0.9573	**0.9753**
	18	0.9788	0.9743	0.9813	0.9831	0.9811	0.9862	0.9731	0.9861	0.9864	0.9861	0.9726	**0.9869**
	22	0.9821	0.9783	0.9837	0.9902	0.9855	0.9911	0.9746	0.9893	0.9900	0.9908	0.9778	**0.9914**
	26	0.9845	0.9829	0.9843	0.9925	0.9910	0.9924	0.9750	0.9908	0.9917	**0.9927**	0.9856	0.9925
Average		0.9527	0.9626	0.9583	0.9655	0.9634	0.9682	0.9520	0.9415	0.9670	0.9675	0.9524	**0.9715**

**Table 15:** The NCC results of satellite image segmentation using hybrid fitness function for all algorithms

Image	*K*	Algorithm											
		RSA	SOA	BWOA	MPA	AO	SMA	AOA	JOA	MFO	HHO	SCA	Proposed COVID
Sat_img1	6	0.9666	0.9700	0.9462	0.9676	0.9644	0.9684	0.9569	0.9727	0.9668	0.9729	0.9652	**0.9749**
	10	0.9784	0.9853	0.9675	0.9850	0.9858	0.9870	0.9854	0.9866	0.9872	0.9875	0.9787	**0.9886**
	14	0.9817	0.9921	0.9897	0.9924	0.9926	0.9938	0.9892	0.9935	0.9939	0.9936	0.9862	**0.9942**
	18	0.9886	0.9945	0.9920	**0.9962**	0.9951	0.9960	0.9910	0.9952	0.9957	0.9957	0.9910	**0.9962**
	22	0.9917	0.9949	0.9945	0.9969	0.9959	0.9967	0.9932	0.9965	**0.9986**	0.9966	0.9917	0.9970
	26	0.9937	0.9960	0.9949	0.9970	0.9967	0.9976	0.9944	0.9971	**0.9978**	0.9975	0.9928	**0.9978**
Sat_img2	6	0.9727	**0.9841**	0.9822	0.9821	0.9804	0.9838	0.9791	0.8894	0.9822	0.9818	0.9781	**0.9841**
	10	0.9730	0.9905	0.9848	0.9902	0.9890	0.9902	0.9842	0.9087	0.9899	0.9901	0.9797	**0.9907**
	14	0.9732	0.9932	0.9865	0.9936	0.9940	0.9934	0.9823	0.9346	0.9935	0.9936	0.9817	**0.9946**
	18	0.9928	0.9943	0.9944	0.9942	0.9937	0.9942	0.9894	0.9825	0.9940	0.9941	0.9847	**0.9949**
	22	0.9937	0.9957	0.9956	0.9958	0.9950	0.9954	0.9943	0.9853	0.9951	0.9957	0.9950	**0.9964**
	26	0.9953	0.9964	0.9948	0.9964	0.9970	0.9965	0.9954	0.9893	0.9963	**0.9974**	0.9962	0.9970
Sat_img3	6	0.9797	0.9821	0.9806	0.9837	0.9850	0.9859	0.9824	0.9852	0.9833	0.9856	0.9817	**0.9865**
	10	0.9871	0.9914	0.9916	0.9881	0.9896	0.9925	0.9901	0.9920	0.9907	0.9920	0.9868	**0.9931**
	14	0.9938	0.9949	0.9943	0.9954	0.9947	0.9960	0.9935	0.9959	0.9943	**0.9963**	0.9883	**0.9963**
	18	0.9952	0.9966	0.9960	**0.9978**	0.9969	0.9972	0.9943	0.9968	0.9975	0.9974	0.9962	0.9977
	22	0.9965	0.9969	0.9968	0.9980	0.9978	0.9981	0.9957	0.9978	0.9981	**0.9983**	0.9968	**0.9983**
	26	0.9974	0.9975	0.9974	0.9986	0.9984	0.9986	0.9960	0.9981	0.9985	**0.9988**	0.9975	**0.9988**
Sat_img4	6	0.9904	0.9913	0.9925	0.9924	0.9922	0.9925	0.9883	0.9925	0.9925	0.9924	0.9920	**0.9928**
	10	0.9934	0.9947	0.9956	0.9961	0.9963	0.9964	0.9897	0.9963	0.9964	0.9963	0.9959	**0.9967**
	14	0.9971	0.9982	0.9978	0.9983	0.9981	0.9985	0.9982	0.9983	**0.9986**	0.9984	0.9968	**0.9986**
	18	0.9979	0.9986	0.9980	0.9987	0.9985	0.9988	0.9982	0.9986	**0.9998**	0.9986	0.9980	0.9992
	22	0.9983	0.9991	0.9984	0.9990	0.9990	0.9994	0.9986	0.9993	**0.9994**	0.9992	0.9988	**0.9994**
	26	0.9988	0.9993	0.9986	0.9993	0.9993	0.9995	0.9989	0.9995	**0.9996**	0.9994	0.9990	**0.9996**
Sat_img5	6	0.9746	0.9763	0.9772	0.9754	0.9758	0.9757	0.9740	0.9768	0.9754	0.9754	0.9638	**0.9787**
	10	0.9844	0.9890	0.9886	0.9898	0.9904	0.9905	0.9852	0.9899	0.9900	0.9905	0.9840	**0.9909**
	14	0.9869	0.9938	0.9919	0.9945	0.9948	0.9948	0.9893	0.9935	**0.9952**	0.9935	0.9902	0.9948
	18	0.9926	0.9946	0.9941	0.9964	0.9958	**0.9967**	0.9920	0.9964	0.9965	0.9962	0.9937	**0.9967**
	22	0.9943	0.9968	0.9955	0.9968	0.9963	0.9970	0.9935	0.9964	**0.9972**	0.9966	0.9942	0.9970
	26	0.9943	0.9965	0.9965	0.9972	0.9973	0.9978	0.9951	0.9916	0.9980	**0.9981**	0.9948	0.9980
Sat_img6	6	0.9783	0.9813	0.9770	0.9812	0.9817	0.9814	0.9795	0.9817	0.9812	0.9828	0.9790	**0.9833**
	10	0.9800	0.9841	0.9829	0.9854	**0.9955**	0.9848	0.9832	0.9850	0.9852	0.9870	0.9866	0.9950
	14	0.9925	0.9940	0.9923	0.9959	0.9961	0.9963	0.9936	0.9961	0.9960	0.9959	0.9890	**0.9965**
	18	0.9948	0.9951	0.9959	0.9974	0.9972	0.9973	0.9952	0.9974	0.9975	0.9972	0.9927	**0.9978**
	22	0.9968	0.9964	0.9966	0.9982	0.9981	0.9983	0.9959	0.9940	0.9982	0.9980	0.9941	**0.9985**
	26	0.9972	0.9969	0.9969	**0.9988**	0.9984	0.9984	0.9963	0.9985	0.9986	0.9987	0.9959	**0.9988**
Average		0.9887	0.9922	0.9901	0.9927	0.9928	0.9932	0.9897	0.9855	0.9930	0.9933	0.9890	**0.9941**

These experiments proved the ability of the proposed algorithm to find the threshold values that most fit segmentation. In terms of the best fitness, it is noticed from Tables 4 and 10 that the proposed algorithm achieved the optimum fitness in 24 from 36 cases for the benchmark images and in 28 from 36 cases for the satellite images. The proposed algorithm produced fitness values very close to the optimum in the remaining cases. The HHO algorithm ranks second after COVID, where it achieved the highest fitness in 8 from 36 cases.

The MSE values in Tables 5 and 11 illustrate that the proposed algorithm has the minimum MSE values in 29 from 36 cases for the benchmark images and 27 for the satellite images. MPA, HHO, and MFO produce results close to the proposed algorithm; however, the proposed algorithm outperforms them significantly. The PSNR is evaluated to measure the power of the segmented image against noise. The PSNR values produced by running all algorithms at different threshold values are shown in Tables 6 and 12 for the benchmark and satellite images.

Regarding PSNR, the proposed algorithm outperforms the other algorithms in 28 from 36 cases for the benchmark images and 30 from 36 cases for the satellite images. Also, the SSIM and FSIM metrics are measured to evaluate the similarity between the original and segmented images. The SSIM results of all algorithms are shown in Tables 7 and 13 for the two datasets. The proposed algorithm is superior in 26 from 36 cases for the benchmark images and 28 from 38 for the satellite images.

According to FSIM, the proposed algorithm is superior in 30 and 29 of 36 cases for the benchmark and satellite images, respectively, as shown in Tables 8 and 14. However, MPA, SMA, and HHO algorithms perform close to the proposed algorithm. The proposed algorithm outperforms them in most the cases.

Finally, the NCC is evaluated to measure the correlation between the original and segmented images. According to the NCC results shown in Tables 9 and 15, it’s clear that the proposed algorithm is superior in 29 and 31 of 36 cases for both datasets. The previous results show that the proposed algorithm's SSIM, FSIM, and NCC values are close to 1, the best possible value. Thus, the proposed algorithm finds the optimum threshold values for image segmentation.

**Fig. 4:**
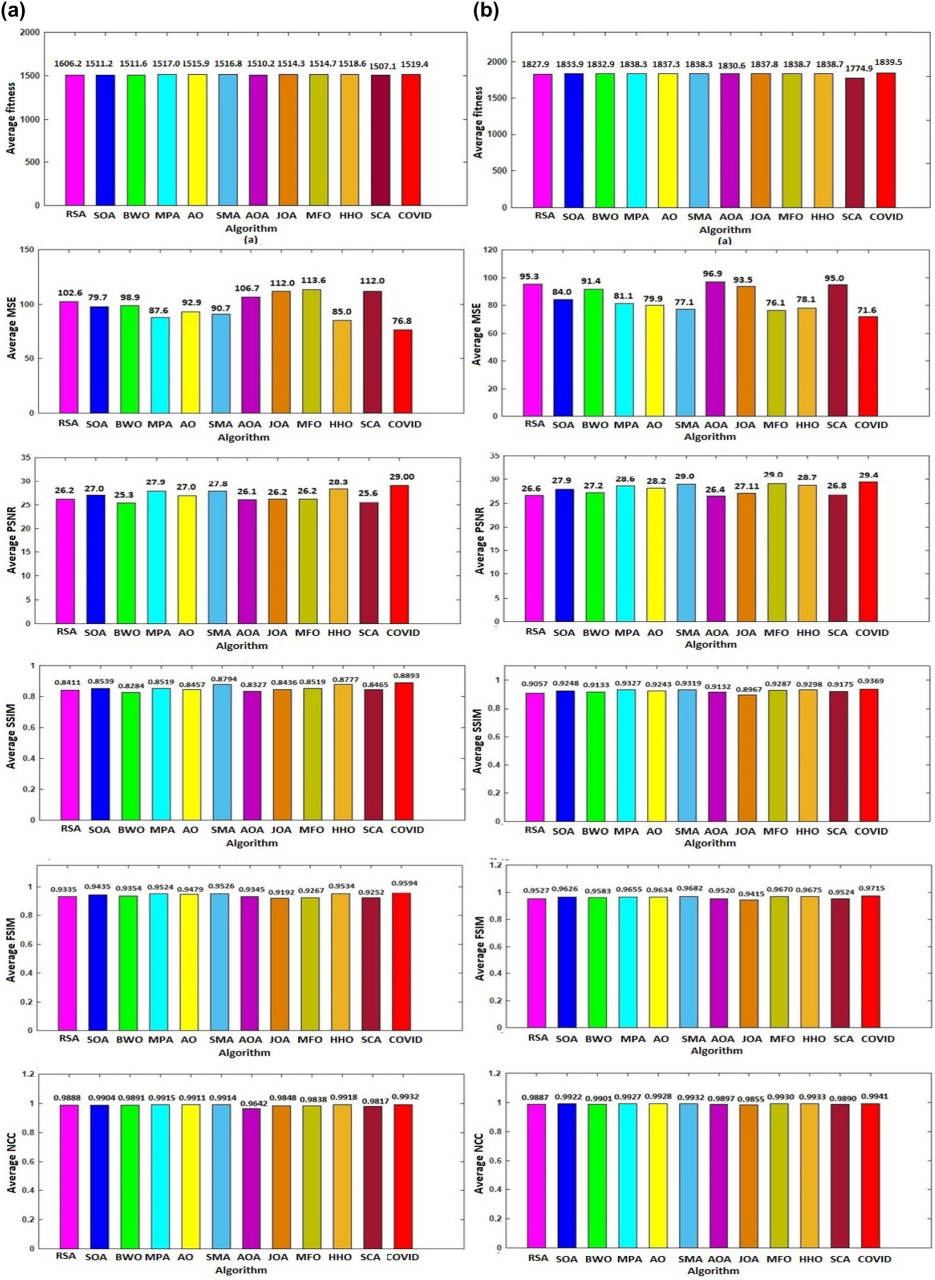
The average fitness, MSE, PSNR, SSIM, FSIM, and NCC results of segmentation of **a** standard images and **b** satellite images for all algorithms

The proposed algorithm is compared to its peers in terms of the total average values for fitness, MSE, PSNR, SSIM, FSIM, and NCC, and the results are shown in Fig. 4. In terms of fitness, the proposed algorithm exceeds all other algorithms, averaging 1519.4 for the standard dataset and 1839.5 for the satellite dataset. However, HHO performs similarly; the proposed algorithm is slightly superior.

As shown in Fig. 4, the proposed algorithm has the minimum total average MSE for both datasets. It is obvious from the figure that there is a clear gap between the average MSE results produced by the proposed algorithm and those produced by the other algorithms. The bar charts for all the six metrics demonstrate that the proposed algorithm is superior. The highest PSNR, SSIM, FSIM, and NCC values achieved by the proposed algorithm demonstrate the high quality of the segmented images produced by the proposed algorithm.

**Fig. 5:**
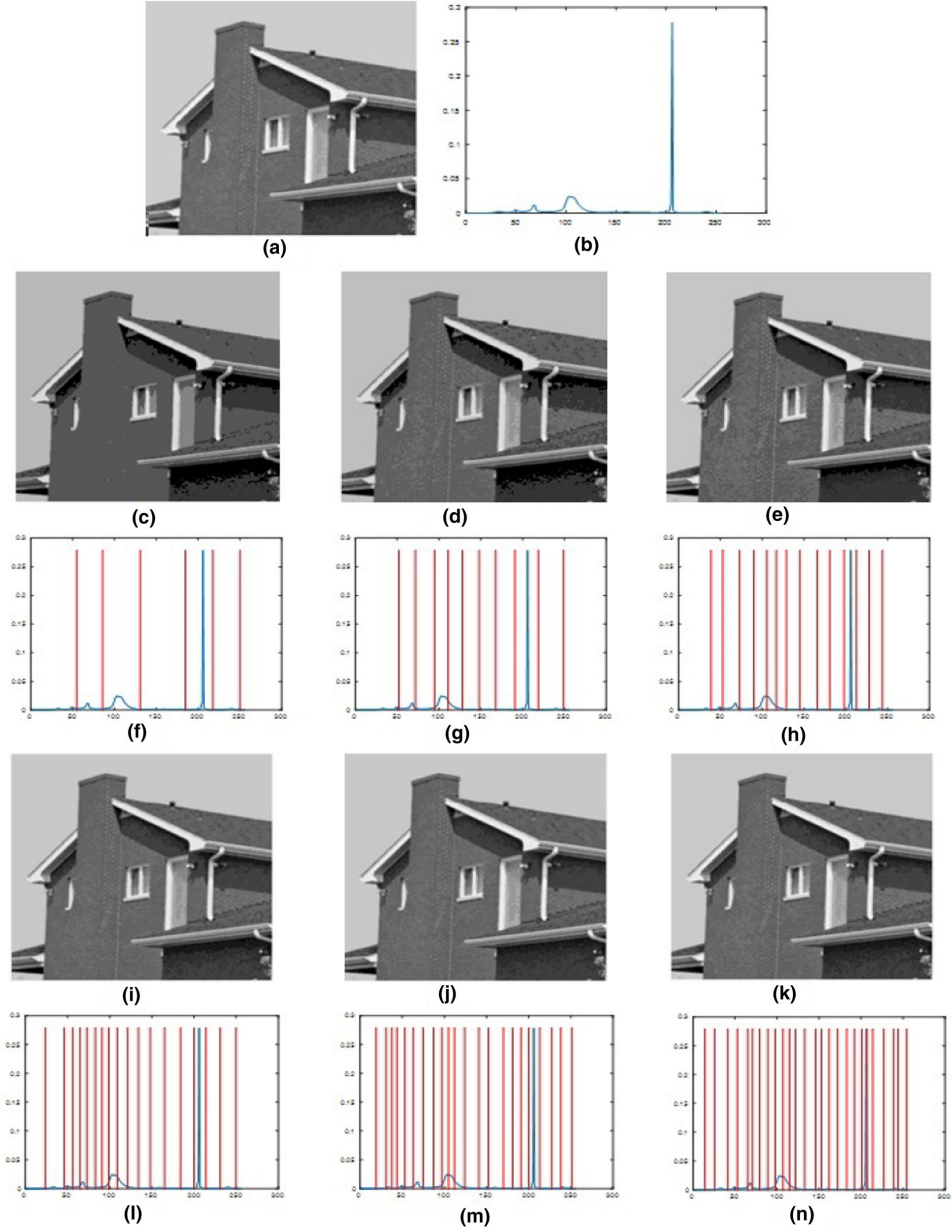
Results for using the proposed algorithm for segmentation of Image2 at different threshold levels. **a** Original image, **b** histogram of the original image, **c**–**e**, **i**–**k** 6-level to 26-level thresholding based segmented images, and **f**–**h**, **l**–**n** 6-level to 26-level corresponding histograms

**Fig. 6:**
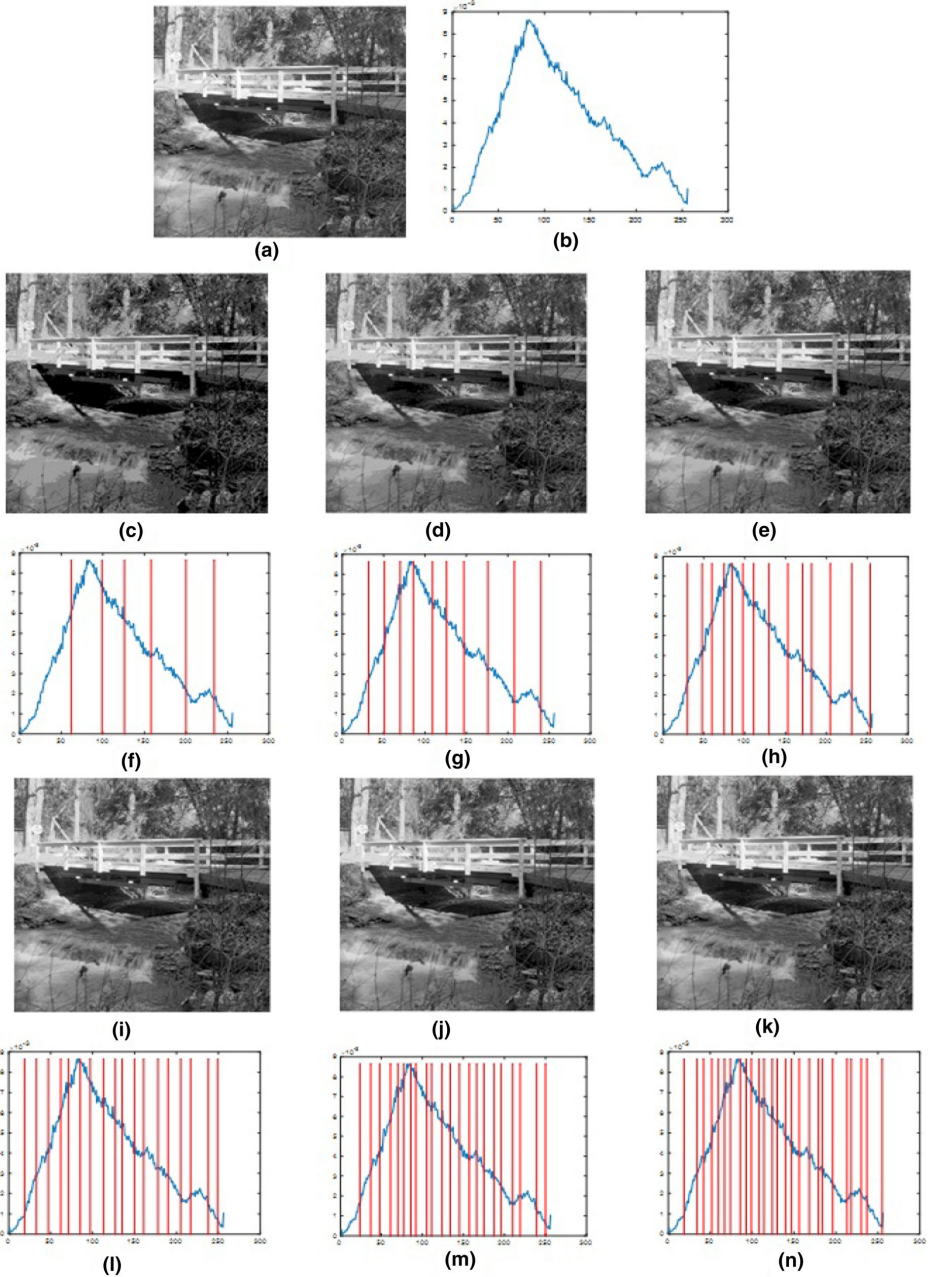
Results for using the proposed algorithm for segmentation of Image4 at different threshold levels. **a** Original image, **b** histogram of the original image, **c**–**e**, **i**–**k** 6-level to 26-level thresholding based segmented images, and **f**–**h**, **l**–**n** 6-level to 26-level corresponding histograms

**Fig. 7:**
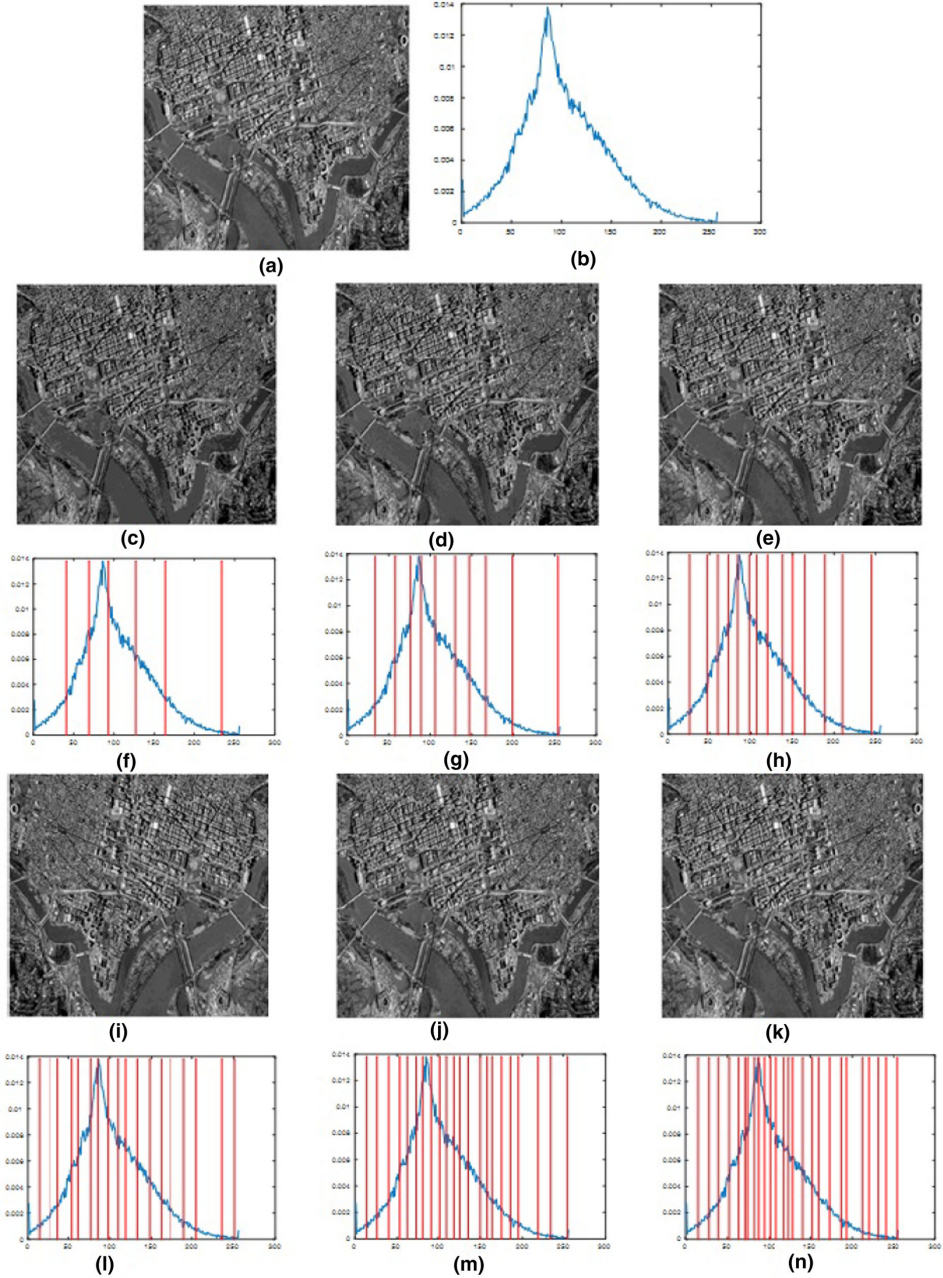
Results for using the proposed algorithm to segment Sat_img1 at different threshold levels. **a** Original image, **b** histogram of the original image, **c**–**e**, **i**–**k** 6-level to 26-level thresholding based segmented images, and **f**–**h**, **l**–**n** 6-level to 26-level corresponding histograms

**Fig. 8:**
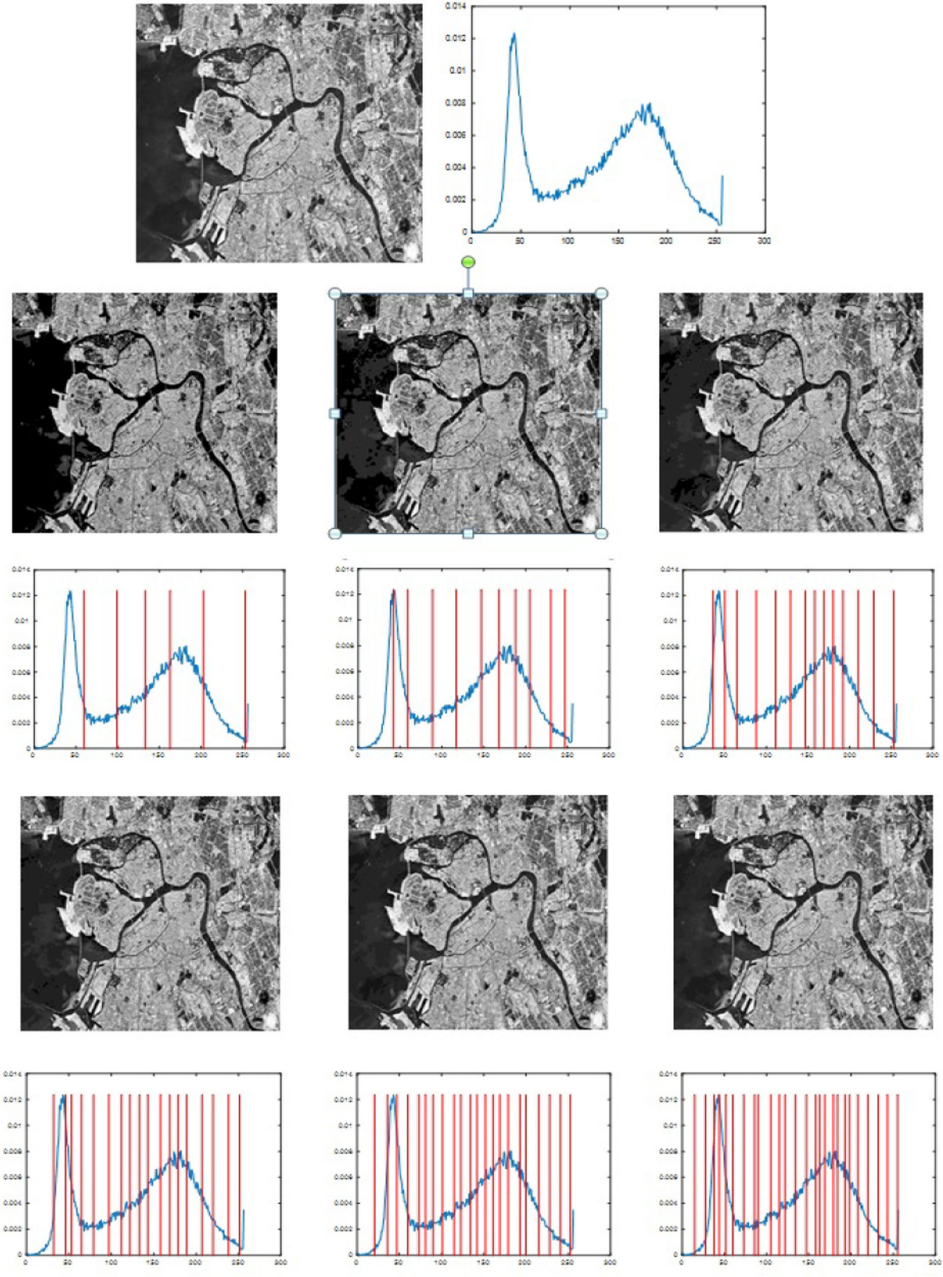
Results for using the proposed algorithm to segment Sat_img3 at different threshold levels. **a** Original image, **b** histogram of the original image, **c**–**e**, **i**–**k** 6-level to 26-level thresholding based segmented images, and **f**–**h**, **l**–**n** 6-level to 26-level corresponding histograms

The segmented images produced by the proposed algorithm at different thresholds are shown in Figs. 5, 6, 7 and 8. The high quality of the segmented images is clear from their visual appearance.

**Fig. 9:**
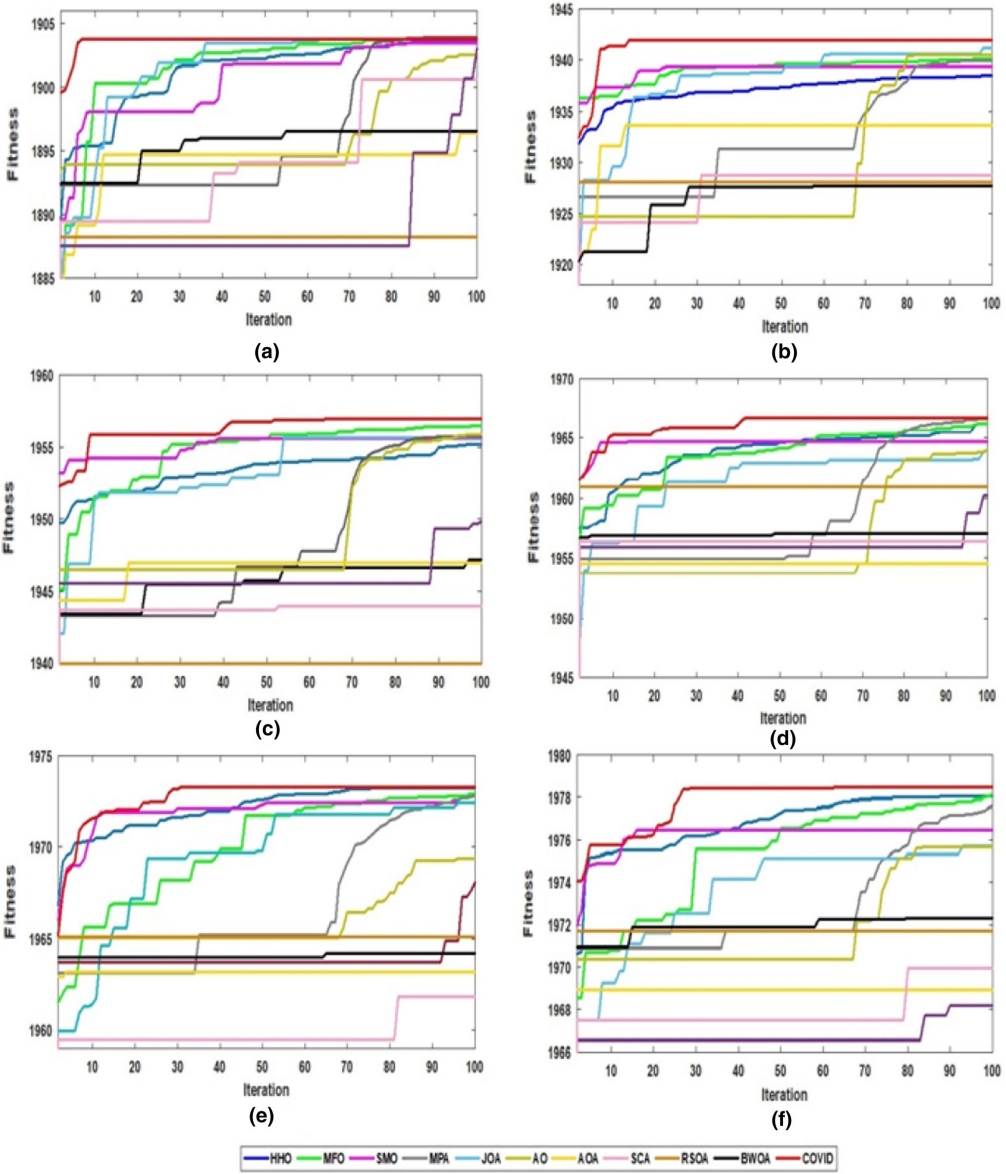
Comparison of convergence curves of all algorithms for segmentation of Sat_img3 with number of thresholds: **a** 6, **b** 10, **c** 14, **d** 18, **e** 22 and **f** 26

Additionally, some convergence curves are displayed in Fig. 9 to show the proposed algorithm's convergence ability. The proposed algorithm has a high convergence rate compared with the other algorithms as it rapidly reaches the highest fitness value.

Due to the random process in optimization algorithms, the results differ at each run. The results of 5 separate runs of the proposed algorithm for segmentation of Image1 and Sat_img1 are shown in Table 16, and the best results are highlighted in bold. However, the results of each run are not the same; they are very close, which ensures the stability of the proposed algorithm.

**Table 16:** The results of 5 different runs of the proposed algorithm for segmentation Image1 and Sat_img1

	Metric	Image1						Sat_img1					
		*K* = 6	*K* = 10	*K* = 14	*K* = 18	*K* = 22	*K* = 26	*K* = 6	*K* = 10	*K* = 14	*K* = 18	*K* = 22	*K* = 26
Run1	Fitness	1899.358	1929.535	1941.70	1949.211	**1955.393**	1959.80	882.077	920.923	**936.4828**	944.831	951.28	956.24
	MSE	153.1303	107.8424	**66.8752**	60.9820	50.6567	**26.3142**	**160.488**	110.516	68.2388	48.1278	34.342	25.384
	PSNR	23.4859	27.1080	**29.7456**	30.1669	31.0628	**33.926**	22.5662	**27.558**	29.9685	31.7148	32.752	34.078
	SSIM	0.6864	0.7430	**0.7855**	0.7949	0.8157	**0.9370**	0.9189	0.9686	**0.9775**	**0.9858**	0.9884	0.9896
	FSIM	0.9180	0.9491	0.9736	0.9783	**0.9843**	0.9852	0.9520	0.9782	**0.9888**	0.9915	**0.9946**	0.9956
	NCC	0.9891	0.9959	**0.9978**	**0.9985**	**0.9987**	**0.9989**	0.9693	0.9874	**0.9935**	0.9955	0.9969	0.9976
Run2	Fitness	1898.815	1929.969	1941.39	1949.752	1954.805	1959.86	882.930	919.595	935.8556	944.983	951.07	956.19
	MSE	162.2831	110.7264	81.6184	57.3263	51.9169	43.8623	166.312	121.541	70.7002	47.1469	38.975	24.503
	PSNR	23.3905	26.9553	28.9696	30.5657	30.8559	31.7088	22.5589	25.9177	**29.8522**	32.1676	32.822	34.238
	SSIM	**0.6993**	0.7533	0.7832	**0.8014**	0.8057	0.8334	0.9198	0.9573	0.9769	0.9825	0.985	0.9906
	FSIM	0.9195	0.9548	0.9674	0.9797	0.9825	0.9855	0.9515	0.9781	0.9883	0.9917	0.9947	**0.9972**
	NCC	0.9900	0.9955	0.9975	0.9984	**0.9987**	0.9987	0.9692	0.9869	0.9930	0.9955	0.9966	0.9975
Run3	Fitness	1899.142	1930.100	1941.60	1949.616	1954.693	1959.41	882.529	920.940	935.6971	944.633	**951.58**	956.17
	MSE	155.2112	111.3618	71.7428	**55.1395**	**50.0202**	27.0324	162.284	112.317	71.7087	50.1352	**30.445**	23.876
	PSNR	23.0967	26.9552	28.9838	**30.8667**	**31.1677**	33.8918	**22.7439**	26.7419	29.2506	32.0419	**33.446**	**34.827**
	SSIM	0.6907	0.7508	0.7772	0.7988	0.8202	0.9399	0.9246	0.9615	0.9756	0.9839	**0.9888**	0.9908
	FSIM	0.9145	0.9566	0.9715	**0.9805**	0.9822	**0.9876**	0.9555	**0.9810**	0.9878	0.9915	0.9944	0.9958
	NCC	0.9878	**0.9960**	0.9978	0.9982	0.9986	0.9988	**0.9722**	**0.9885**	0.9930	0.9952	**0.9972**	**0.9978**
Run4	Fitness	1899.576	**1930.926**	1942.03	1949.275	1954.997	**1960.01**	882.767	921.304	936.1029	**945.085**	951.17	**956.63**
	MSE	156.4345	**102.8089**	78.5719	60.2595	50.1078	28.0079	161.988	113.127	**66.2915**	51.0218	32.949	25.746
	PSNR	23.2773	**27.6671**	29.0128	30.1015	31.1047	33.6565	22.3728	26.8638	29.3662	32.0259	32.694	34.023
	SSIM	0.6894	0.7620	0.7777	0.7955	0.8119	0.9344	**0.9254**	0.9579	0.9755	0.9827	0.9878	0.9910
	FSIM	0.9144	**0.9601**	**0.9745**	0.9798	0.9818	0.9867	0.9496	0.9776	0.9882	0.9918	0.9945	0.9956
	NCC	0.9887	**0.9960**	0.9976	0.9984	0.9986	0.9988	0.9684	0.9878	0.9934	0.9956	0.9967	0.9976
Run5	Fitness	**1899.789**	1929.897	**1942.30**	**1950.106**	1955.312	1959.80	**882.981**	**921.344**	935.2058	944.915	951.10	956.14
	MSE	**152.1230**	106.5899	75.6387	58.0894	51.3986	29.0113	164.264	**107.302**	67.7529	**45.6485**	32.360	**22.758**
	PSNR	**23.8876**	26.9943	28.9731	30.4123	31.0033	33.7049	22.5806	26.6298	29.6812	**32.1921**	33.116	34.192
	SSIM	0.6947	0.**7655**	0.7825	0.7987	**0.8209**	0.9339	0.9215	**0.9692**	0.9768	0.9824	0.9885	**0.9915**
	FSIM	**0.9220**	0.9575	0.9732	0.9801	0.9821	0.9864	**0.9567**	0.9768	0.9855	**0.9935**	0.9942	0.9954
	NCC	**0.9906**	0.9959	**0.**9977	0.9983	0.9985	0.9988	0.9718	0.9876	0.9925	**0.9958**	0.9968	**0.9978**

In addition to the previously mentioned evaluation criteria, the Wilcoxon rank-sum test is utilized to prove the statistical significance of the proposed algorithm. This test compares two methods based on the null hypothesis, which assumes no significant difference between the two methods. The *P* values produced by the Wilcoxon rank-sum test must be ≤ 0.05 to be good evidence against the null hypothesis.

The *P* values produced by comparing the proposed algorithm with all other algorithms are shown in Tables 17 and 18. All the *P* values shown in the table are ≤ 0.05, which proves the alternative hypothesis that assumes a significant difference between the two methods. The overall results prove the efficiency of the proposed algorithm in image segmentation.

**Table 17:** The *P* values computed by Wilcoxon's rank-sum test for segmentation of benchmark images

Benchmark images										
Image	COVID versus RSOA	COVID versus SOA	COVID versus BWOA	COVID versus AO	COVID versus AOA	COVID versus JOA	COVID versus SMO	COVID versus MFO	COVID versus HHO	COVID versus SCA
Image1	1.9365e−42	1.0942e−40	5.0076e−39	1.1849e−13	4.1998e−40	4.4888e−14	4.7950e−21	7.5760e−16	5.1721e−06	7.8921e−37
Image2	2.8361e−41	2.2067e−39	4.3256e−42	5.9331e−09	5.2181e−42	1.6584e−22	2.7321e−16	5.7751e−21	4.8709e−08	2.9425e−38
Image3	1.4777e−44	1.7535e−36	3.2046e−42	3.3224e−14	7.0377e−42	9.3245e−09	2.4728e−13	5.0899e−13	9.9852e−06	2.4780e−40
Image4	9.1065e−44	1.2034e−40	3.2241e−39	1.1358e−09	2.1267e−43	4.2730e−08	1.0263e−16	9.0875e−21	3.1708e−10	7.5621e−39
Image5	1.4777e−44	5.8168e−38	2.5466e−39	4.8209e−09	3.1631e−43	1.7981e−12	1.420e−20	5.8680e−23	1.3722e−13	6.2710e−37
Image6	2.2404e−40	1.2704e−40	4.0104e−39	1.1946e−07	2.5718e−42	2.621e−16	8.1879e−15	3.3620e−13	1.1222e−10	1.1783e−36
Average	**4.24e−41**	**3.02e−37**	**2.47e−39**	**7.19e−08**	**7.26e−41**	**8.68e−09**	**4.26e−14**	**1.41e−13**	**2.53e−06**	**4.39e−37**

**Table 18:** The *P* values computed by Wilcoxon's rank-sum test for segmentation of satellite images

Satellite images										
Image	COVID versus RSOA	COVID versus SOA	COVID versus BWOA	COVID versus AO	COVID versus AOA	COVID versus JOA	COVID versus SMO	COVID versus MFO	COVID versus HHO	COVID versus SCA
Sat_img1	3.2950e−39	9.6055e−39	6.7688e−34	8.8471e−07	6.0305e−36	0.00276	4.1448e−04	4.0816e−14	1.7974e−12	3.5241e−36
Sat_img2	2.1450e−38	2.8740e−33	5.6072e−35	4.9599e−12	4.1518e−22	7.3150e−05	1.4589e−07	3.7710e−11	2.9632e−11	1.9025e−35
Sat_img3	1.1344e−38	3.3244e−36	9.3573e−32	2.1921e−22	1.8013e−34	1.0675e−06	6.1799e−04	1.0844e−15	1.0851e−13	5.9112e−35
Sat_img4	6.2761e−36	5.9516e−32	6.7688e−34	1.0167e−21	1.1419e−29	1.9288e−05	2.1584e−06	9.3069e−19	2.7708e−10	3.5221e−34
Sat_img5	4.2923e−33	2.1586e−35	2.1698e−34	6.2436e−18	6.7921e−39	2.5615e−05	5.4034e−08	1.2381e−10	1.6474e−11	1.8610e−34
Sat_img6	5.2142e−34	4.2254e−36	1.8022e−35	4.1430e−20	2.3102e−36	1.3020e−06	2.1694e−12	5.3655e−14	1.8087e−13	1.6125e−35
Average	**8.03e−34**	**1.04e−32**	**1.59e−32**	**1.47e−07**	**6.92e−23**	**4.80e−04**	**1.72e−04**	**2.69e−11**	**5.42e−11**	**1.06e−34**

## Conclusions and future work

Satellite image segmentation aims to get a map composed of a few categories (buildings, roads, tracks, trees, crops and water, etc.) from a multispectral satellite image in many applications such as geoscience studies, astronomy, and geographical information systems. This paper proposes an improved Coronavirus Disease Optimization algorithm for solving satellite image's multi-level thresholding segmentation problem. The concept of chaotic initialization is embedded into the proposed algorithm to improve the searchability of the initial population and to void the problem of getting stuck into local minima or maxima. Additionally, a hybrid fitness function is utilized to measure the fitness of solutions instead of the classic Otsu and Kapur methods. Two separate datasets are segmented using the proposed algorithm, and several evaluation criteria have been utilized to measure the performance. The experimental results proved that the proposed algorithm with chaotic initialization and the hybrid fitness function results in image segmentation with better performance than other metaheuristics. Future work will apply the proposed algorithm to image segmentation of color images.
